# Protein Hydrolysates and Bioactive Peptides as Mediators of Blood Glucose—A Systematic Review and Meta-Analysis of Acute and Long-Term Studies

**DOI:** 10.3390/nu16020323

**Published:** 2024-01-22

**Authors:** Arig Elbira, Maryam Hafiz, Alan Javier Hernández-Álvarez, Michael A. Zulyniak, Christine Boesch

**Affiliations:** 1 School of Food Science and Nutrition, Faculty of Environment, University of Leeds, Leeds LS2 9JT, UK; fs19aaae@leeds.ac.uk (A.E.); mshafiz@kau.edu.sa (M.H.); a.j.hernandezalvarez@leeds.ac.uk (A.J.H.-Á.); m.a.zulyniak@leeds.ac.uk (M.A.Z.); 2 Department of Clinical Nutrition, Faculty of Applied Medical Sciences, King Abdul-Aziz University, P.O. Box 80215, Jeddah 21589, Saudi Arabia

**Keywords:** bioactive peptides, protein hydrolysates, glucose, diabetes, postprandial glycaemia, systematic review, meta-analysis

## Abstract

Type 2 diabetes mellitus (T2DM) is a major public health concern associated with high mortality and reduced life expectancy. Since diabetes is closely linked with lifestyle, not surprisingly, nutritional intervention and increased physical activity could play a vital role in attenuating the problems related to diabetes. Protein hydrolysates (PHs) and their bioactive peptides (BP) have been shown to exert a wide range of biological effects, including antioxidative, antihypertensive, and in particular, hypoglycaemic activities. To better understand the efficacy of such interventions, a systematic review and meta-analysis of randomised controlled trials (RCTs) were performed concerning the influence of protein hydrolysates on glycaemic biomarkers in subjects with and without hyperglycaemia. Five different databases were used to search for RCTs. In total, 37 RCTs were included in the systematic review and 29 RCTs in the meta-analysis. The meta-analysis revealed a significant reduction in postprandial blood glucose response (PPGR) in normoglycaemic (−0.22 mmol/L; 95% CI −0.43, −0.01; *p* ≤ 0.05) and in hyperglycaemic adults (−0.88 mmol/L; 95% CI −1.37, −0.39; *p* ≤ 0.001) compared with the respective control groups. A meta-regression analysis revealed a dose-dependent response for PPGR following PH consumption in normoglycaemic adults, specifically for doses ≤ 30 g. The postprandial blood insulin responses (PPIR) were significantly higher after the ingestion of PHs in both the group with and the group without hyperglycaemia, respectively (23.05 mIU/L; 95% CI 7.53, 38.57; *p* ≤ 0.01 and 12.57 mIU/L; 95% CI 2.72, 22.41; *p* ≤ 0.01), compared with controls. In terms of long-term responses, there was a small but significant reduction in both fasting blood glucose (FBG) and fasting glycated haemoglobin (HbA1c) in response to PH compared with the control group (*p* < 0.05). The PHs significantly improved the parameters of glycaemia in adults and, hence, it may contribute to the management and regulation of the future risk of developing T2DM.

## 1. Introduction

Type 2 diabetes mellitus (T2DM) is a chronic metabolic disorder characterised by hyperglycaemia due to defective insulin production and/or action [1,2]. It is a common condition, with an estimated worldwide prevalence of 536.6 million people in 2021, and a projected increase to 783.2 million by 2035 [3].

Different types of enzymes are involved in regulating hyperglycaemia, such as α-glucosidase, α-amylase, and dipeptidyl peptidase IV (DPP-IV), and these are released by differing organs (salivary glands, the pancreas, and the small intestine) in the digestive tract [4,5]. Specifically, α-Glucosidase and α-amylase enzymes are involved in the process of carbohydrate hydrolysis, resulting in the release of glucose from starch and disaccharides and, hence, in increased blood glucose levels. Furthermore, DPP-IV plays a major role in glucose metabolism through the rapid degradation of incretins, such as glucagon-like peptide GLP-1, which is the main hormone that helps the body to increase glucose-dependent insulin secretion from the pancreas when required [4].

In terms of pharmacological therapy, several drugs are approved to lower high blood glucose levels. The group of α-glucosidase inhibitors, such as acarbose, voglibose, and miglitol, effectively reduces postprandial glucose levels. However, these inhibitors tend to cause gastrointestinal problems, such as diarrhea, nausea, bloating, abdominal pain, and flatulence. In addition, GLP-1 receptor agonists and DPP-IV, which increase the half-life of GLP-1, are also effective for lowering serum glucose levels, albeit at a high cost and with adverse effects, such as increasing the risk of kidney injury [6].

Nevertheless, based on the National Diabetes Statistic Report of USA (2017), two thirds of T2DM patients suffer from other chronic diseases, such as coronary heart disease, hypertension, peripheral artery disease, and kidney diseases, which might be worsened by drug use, especially when taken on a permanent basis [7]. Therefore, in recent years, a considerable interest has been devoted to the identification and development of food-derived antidiabetic agents that are considered natural and safe with minimal side effects. There is increasing evidence from in vitro studies demonstrating that protein-derived bio-active peptides (BP) and protein hydrolysates (PHs) could provide natural inhibitors for some digestive enzymes and, hence, that they could significantly contribute to controlling blood glucose levels [8,9].

The hydrolysis of proteins generates protein hydrolysates, and the outcome varies based on the specificity of the protease and hydrolysis conditions, such as the enzyme/substrate ratio, pH, temperature, enzyme activity, and length of hydrolysis. This process produces a blend of protein fragments, ranging in size, length, and amino acid composition, and comprising oligopeptides, peptides, and free amino acids. Within this mix, there are bioactive peptides, many of which exhibit biological activities. These BPs are specific protein fragments (typically 2–30 amino acids long) released from the parent protein and exert beneficial effects on bodily functions and/or positively influence human health beyond their known nutritional value. In addition to enzymatic hydrolysis, BPs can also be generated by enzymatic hydrolysis and food processing, as well as microbial fermentation [10].

Furthermore, BPs have a wide range of biological activities in the metabolic functions of living organisms and, consequently, in human health, including antimicrobial, antihypertensive, hypoglycaemic activity, immunomodulatory, and antioxidative effects [11,12].

Numerous in vitro studies have reported the potential beneficial effects of these peptides on blood glucose management, acting through different mechanisms. Mohanty et al. [13] successfully identified and isolated BPs in milk with strong in vitro α-amylase inhibitory activity. Moreover, NVLQPS and KLPGF are BPs derived from albumin that have shown significant activity against α-glucosidase, with almost similar half-maximal inhibitory concentrations (IC_50_ values) to that of the therapeutic drug acarbose [14]. Hence, they may effectively reduce hyperglycaemia in humans. Moreover, in diabetic rats, the absorption of glucose and postprandial glucose levels were reduced after oral ingestion of isolated peptides derived from black beans, which was due to the inhibition of DPP-IV [15].

There is increasing evidence from human intervention studies investigating the biological activity of these BPs in vivo. For example, a study on hyperglycaemic individuals reported a reduction in the area under the curve of the postprandial glucose response after the consumption of milk-protein hydrolysates when compared to placebo, and with a minor postprandial effect on insulin [16]. However, a longer intervention period with similar doses did not strengthen the acute glucose or insulin response, but it did significantly improve long-term factors such as fasting glycated haemoglobin (HbA1c). In contrast, the consumption of marine PHs before a breakfast meal reduced postprandial insulin secretion in healthy adults, albeit without affecting the blood glucose response [17]. The aims of this systematic review and meta-analysis were to identify the reasons for the variation between these studies, to identify the effective dose and conditions and, ultimately, to aid the development of functional products. To the best of our knowledge, this is the first systematic review and meta-analysis to summarise evidence on the acute and long-term effects of PHs on glycaemia in humans.

## 2. Methods

This systematic review and meta-analysis was conducted according to the guidelines of Preferred Reporting Items for Systematic Reviews and Meta-Analyses (PRISMA) [18]. The systematic review was prospectively registered with PROSPERO (CRD42020201551).

### 2.1. Data Source and Search Strategy

The electronic search was conducted on PubMed, Web of Science, Scopus, ScienceDirect, and Cochrane databases and included all materials up to October 2023 based on predefined search terms alongside various Boolean terms (Table S1). Manual search was conducted by reviewing reference lists of identified reviews and included studies.

### 2.2. Study Selection

The ‘title and abstract’ and ‘full text’ screening was performed in duplicate by two independent reviewers. For inclusion, studies were required to be (i) randomised controlled trials (RCTs), either acute (assessing single meal response) or long-term (assessing intake >2 weeks), investigating the effect of food-derived BPs and/or PHs on glycaemia, (ii) in human adult populations, and (iii) published in English. No restrictions were made regarding the health condition, except type 1 diabetes mellitus and gestational diabetes, year of publication, method for obtaining peptides and/or hydrolysates, dosage, administration method, and duration of intervention. In studies in which different interventions were used in different arms, only data from arms that met the eligibility criteria were included in the analysis. Corresponding authors were contacted in cases in which the full data were not available online before deciding on exclusion.

### 2.3. Data Extraction

After identifying all studies to be included in the systematic review, the following data were extracted: year of publication; country of origin; design of the study; number of visits in acute studies; study duration in long-term trials; protein sources; dose; type of control; sample size and participant characteristics (gender, age, BMI, and health condition). Effect sizes were also obtained, mainly in the form of means and variance (standard deviation, standard error, and confidence interval). Baseline and postprandial glucose response (PPGR, mmol/L) and postprandial insulin response (PPIR, mIU/L) values and their areas under the curves (AUCs) were extracted from acute studies. For long-term trials, data extraction included fasting blood glucose (mmol/L), insulin (mIU/L), HbA1c and homeostatic model assessment of insulin resistance (HOMA-IR). For cases in which data were presented in graph format only, numerical data were extracted using WebPlotDigitizer (v4.5). When extracted data were presented as different units for the same outcome, these values were converted to similar units to use the mean differences for determining the effect sizes. In a manner similar to the screening process, the data extraction was conducted in duplicate by two independent reviewers.

### 2.4. Assessment for Risk of Bias

A modified Cochrane Collaboration’s tool (RoB2) was used to assess the risk of bias for all included RCTs [19]. The retrieved studies were evaluated based on six domains of bias (selection bias, performance bias, detection bias, attrition bias, reporting bias, and other bias). For each domain, different aspects of trial design, conduct, and reporting were assessed as sub-signalling questions, and these were categorised in terms of their potential bias as either ‘Low’, ‘High’, or ‘Some Concerns’.

The decision as to which were the most important domains to be predominantly assessed was made based on the review protocol and PICO model, such as comparability of study groups, method of handling withdrawals, and the use of blinding. After the judgment was made within the trials, a conclusion as to the overall risk of bias across all studies was drawn, and a justification provided. Publication bias was visually assessed by inspection of funnel plots.

### 2.5. Data Analysis

Comprehensive Meta Analysis version 4 (CMA) and Review Manager (RevMan) version 5.4 (Cochrane Informatics and Knowledge Management Department) for Windows 10 were used for different analyses. Assuming there would be variation in the studies, the random effect and weighting in determining pooled estimates were used (DerSimonian and Laird). For publication bias, funnel plots were used to investigate the asymmetry.

The entered data included sample size, as well as reported means and standard deviations for intervention arms and their matched controls for each trial. Studies were presented as a summary table and forest plots. Pooled random-effects analyses using mean difference model were initially performed to estimate the effect size and 95% CI of postprandial glucose and insulin responses in acute RCTs. Analysis of studies based on participants’ baseline glucose statuses was only performed for acute studies, and separated into interventions in normoglycaemic (<5.6 mmol/L) and hyperglycaemic (≥5.6 mmol/L) individuals. Due to the small number (<10) and insufficient data, this could not be applied to long-term studies. Further subgroup analyses were performed for acute studies by type of control, BMI, age, and dose. A negative effect size indicated favouring intervention, whereas a positive effect size referred to favouring control if the confidence interval did not include or cross one and, hence, sufficient evidence was provided to conclude that the groups were statistically significantly different. Heterogeneity among studies was assessed by the χ^2^ test for the Cochran’s Q and *I*^2^ statistics, an estimate of the proportion of variance explained by between-study heterogeneity. An *I*^2^ value less than 50% represented a non-substantial level of heterogeneity. Prediction intervals (PIs) were also calculated to (i) predict the results of a future study with population demographics similar to those in the analyses and (ii) to summarise the spread of underlying effects in the studies included in the analysis. Meta-regression was considered in case of *I*^2^ values higher than 50% [20].

### 2.6. Grading the Evidence

An overall GRADE quality rating (Grading of Recommendations Assessment, Development and Evaluation) was applied to summarise certainty of evidence (GRADEpro, 2020). The GRADE rating has four levels of evidence: very low, low, moderate, and high. The level of certainty was determined based on five different domains: risk of bias, imprecision, inconsistency, indirectness, and publication bias.

## 3. Results

**Figure 1:**
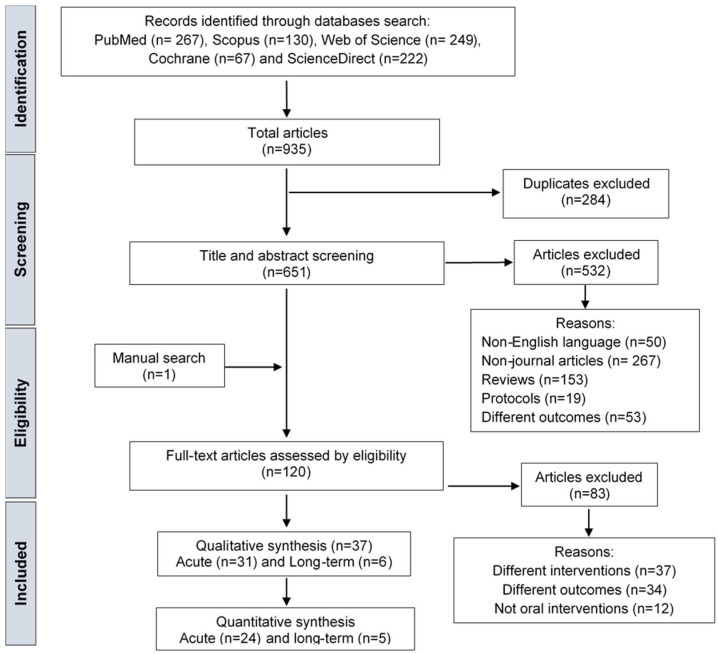
PRISMA flow diagram outlining the identification, screening, and selection of studies that were included in the systematic review.

The initial search of the databases resulted in a total of 935 studies, from which, after removing the duplicates, 651 were screened based on title and abstract (see PRISMA flowchart, Figure 1). In addition to the 119 that passed the title-and-abstract screening, one article was added manually from the references, which provided 120 articles for full-text screening. Of these, 83 studies were excluded for not meeting the inclusion criteria. In total, 37 studies fulfilled the eligibility criteria. After considering articles with multiple arms, 42 comparisons (within the 37 studies) were classified, according to baseline glucose status, in the normoglycaemic group (Table 1) or the hyperglycaemic group (Table 2).

A risk of bias was evident in most of the RCTs due to insufficient details on the randomisation concealment and the selection of the reported outcomes (see Supplementary File, Table S2). Among the RCTs, seven were classified as ‘high-risk’ due to concerns in three or more domains. This was mainly due to bias in the selective reporting of the outcomes or incomplete reporting of the study methods. One study was also assessed as ‘high-risk’ as it was published over two decades ago and, considering the evolving standards in RCT reporting during that period, the study was not excluded from the meta-analysis.

### 3.1. Parameters of Acute Postprandial RCTs

**Figure 2:**
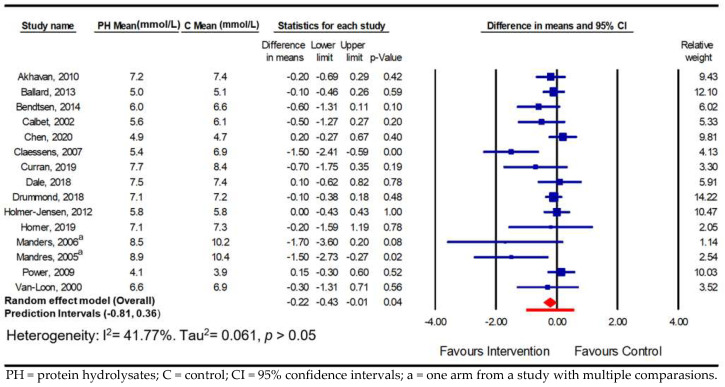
Pooled effect using inverse-variance random-effect model (mean difference and 95% CI) of acute trials investigating effects of oral doses of protein hydrolysates on postprandial glucose response among normoglycaemic adults [17,22,23,24,25,26,28,30,32,33,34,36,37,39,40].

**Figure 3:**
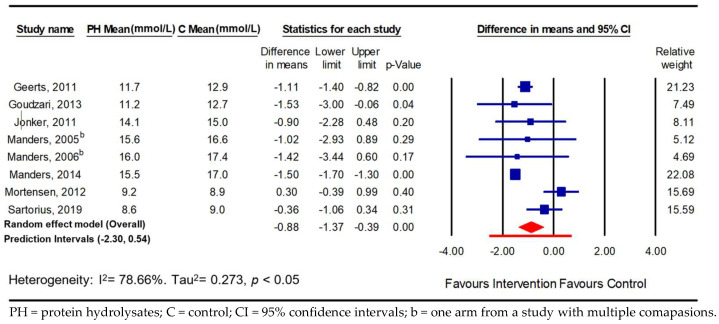
Pooled effect using inverse-variance random-effect model (mean difference and 95% CI) of acute trials investigating effects of oral doses of protein hydrolysates on postprandial glucose response among hyperglycaemic adults [16,36,37,44,45,47,50,51].

The meta-analysis showed that a dose of protein hydrolysate significantly improved the parameters of postprandial glycaemic handling. Overall, there was a significant reduction in PPGR in the normoglycaemic adults compared with the control group in 15 comparisons (−0.22 mmol/L; 95% CI −0.43, −0.01; *p* ≤ 0.05; *I*^2^ = 41.7%; PI −0.81, 0.36) and in eight comparisons in adults with hyperglycaemia (−0.88 mmol/L; 95% CI −1.37, −0.39; *p* ≤ 0.001; *I*^2^ = 78.7%; PI −2.30, 0.54) (Figure 2 and Figure 3), with high heterogeneity observed in the hyperglycaemia analysis. When expressed as a prediction interval, the spread of underlying effects in the studies and uncertainty in future study results can be appreciated and encourages researchers undertaking subgroup analyses to identify study- and population-level mediators of efficacy.

**Figure 4:**
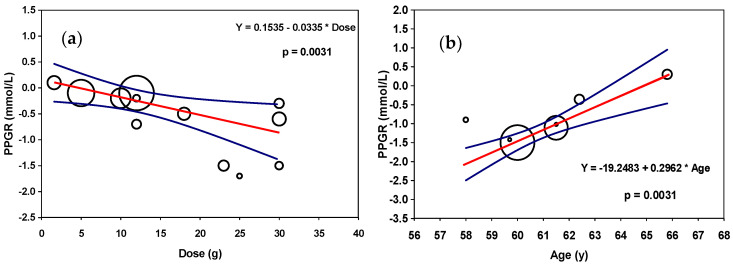
Random-effects meta-regression on the impact of (**a**) protein-hydrolysate dose on PPGR reduction in normoglycaemic adults and (**b**) age on PPGR in hyperglycaemic adults. The size of the bubbles is proportional to the weight that the studies received in the meta-analysis. The true estimate effect is shown as a red line with corresponding 95% confidence interval bounds as blue curved lines.

The subgroup analysis based on different intervention doses in the normoglycaemic individuals revealed a dose-dependent increase in PPGR up to 30 g, which was only significant for the groups administered 21–30 g (−0.97 mmol/L; 95% CI −1.50, −0.44; *p* ≤ 0.001) but not for those administered ≤10 g (−0.1 mmol/L) and 10–20 g (−0.2 mmol/L) (Figure S1). In contrast, the interventions of over 30 g showed a mean increase of 0.11 mmol/L (*p* > 0.05). In addition, dividing the studies according to dose significantly reduced overall the heterogeneity between the studies from 42% to 25% in the 21–30 g group. In the meta-regression, a dose–response relationship was only confirmed between the PPGR and PH interventions in the adults with normal blood glucose levels (*p* ≤ 0.01) who received a dose of up to 30 g (Figure 4a). A potential reason for the lack of correlation in the very-high-dose group may be the use of whey isolates as the controls compared to the lower-dose groups, in which casein was more commonly used.

The subgroup analysis conducted by grouping the control foods suggested that the reduction in PPGR in response to PHs was only significant when placebos (carbohydrates) were used as controls (−0.51 mmol/L; 95% CI −1.02, −0.01; *p* ≤ 0.05; *I*^2^ = 72%) compared to protein isolate (*p* > 0.05) in healthy adult RCTs (Figure S2). Moreover, the subgroup analysis conducted by using the participants’ BMI did not reduce the heterogeneity in the normoglycaemic adults. However, there was an increase in PPGR reduction to −0.30 mmol/L in the group with BMIs of more than 25 kg/m^2^ (*p* ≤ 0.05) (Figure S3). Although not to a significant degree, the subgroup analysis by age strengthened the PPGR in two out of the three subgroups (18–35 y, −0.33 mmol/L, and ≤56, −0.66 mmol/L) (Figure S4).

In the hyperglycaemic category, the subgroup analysis, which included eight of the acute intervention trials, revealed significant PPGR reductions of −0.93 mmol/L (95% CI −1.37, −0.50; *p* ≤ 0.001) in the group receiving ≤20 g, with a significant change in heterogeneity, from 79% to 30% (Figure S5). However, the sensitivity analysis of the BMI and type of control improved the heterogeneity between the studies only marginally (Figures S6 and S7), with no significant change found in terms of age (Figure S8). A further meta-regression analysis revealed a significant positive association between age and PPGR in response to PHs in the hyperglycaemic subjects, which could partially explain the high level of heterogeneity between the studies (*p* ≤ 0.001) (Figure 4b). However, no significant correlations were found between the PPGR and any other co-variates, such as dose and BMI.

**Figure 5:**
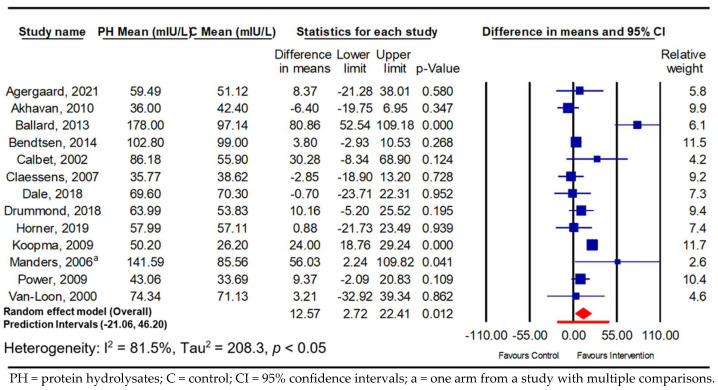
Pooled effect using inverse-variance random-effect model (mean difference and 95% CI) of acute trials investigating the effect of oral doses of protein hydrolysates on postprandial insulin response among normoglycaemic adults [17,21,22,23,24,25,28,32,34,35,37,39,40].

**Figure 6:**
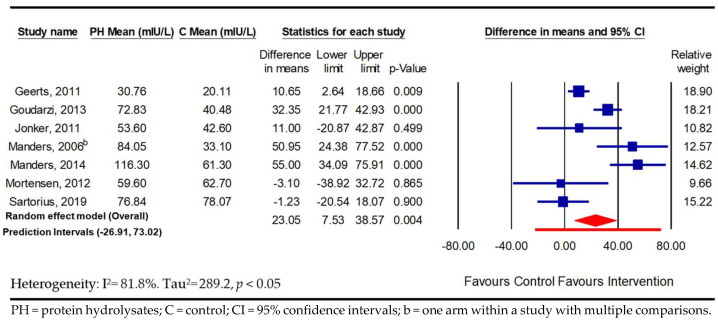
Pooled effect using inverse-variance random-effect model (mean difference and 95% CI) of acute trials investigating effect oral doses of protein hydrolysates on postprandial insulin response among hyperglycaemic adults [16,37,44,45,47,50,51].

In terms of PPIR, significantly increased insulin values were observed in both the adults with and those without hyperglycaemia (*n* = 7; 23.05 mIU/L; 95% CI 7.53, 38.57; *p* ≤ 0.01; *I*^2^ = 81.8%; PI −26.91, 73.02, and *n* = 13; 12.57 mIU/L; 95% CI 2.72, 22.41; *p* ≤ 0.01; *I*^2^ = 81.5%; PI −21.06, 46.20) in response to PH supplementation (Figure 5 and Figure 6). Unlike the PPGR, the subgroup analysis within the normoglycaemic studies according to control type showed that the increase in PPIR was only significantly different when the PH consumption was compared against protein isolate (unhydrolysed) as a control (10.70 mIU/L; 95% CI 0.83, 20.56; *p* ≤ 0.05, *I*^2^ = 78%) (Figure S9). Morover, the subgroup analysis using the participants’ BMI revealed that only the normoglycaemic adults with high BMIs (more than 25 kg/m^2^) had significant increases in PPIR in response to PH (25.06 mIU/L; 95% CI 1.75, 48.37; *p* = 0.04; *I*^2^ = 87%), with a small and non-significat increase in PPIR in the normal BMI group (≤25 kg/m^2^; 7.58 mIU/L; *p* > 0.05) (Figure S10). Another subgroup analysis by dose showed a tendency toward a significant increase in PPIR when higher doses of PH (<20 g) were consumed by the normoglycaemic subjects (10.72 mIU/L; *p* = 0.06) with no improvement in heterogeneity (Figure S11). However, except for the stratification by participant age (Figure S12) and the resulting low level of heterogeneity in the youngest group (18–35 years; *I*^2^ = 6%), the heterogenity remained signficant in all the other subgrop analyses (36–55 and >56 years) (Figure S12).

In terms of the hyperglycaemic group, the subgroup analysis according to dose showed that the increase in PPIR was only significantly different when >20 g PH was consumed (37.13 mIU/L; 95% CI 5.76, 68.57; *p* ≤ 0.05, *I*^2^ = 75%) (Figure S13). There was also a trend towards a significant change in the low-dose group (≤20 g; *p* = 0.06). The sensitivity analysis based on the type of control, BMI, or age did not reveal a meaningful effect on the change in PPIR or measures of heterogeneity (Figures S14–S16).

### 3.2. Parameters of Long-Term RCTs

**Figure 7:**
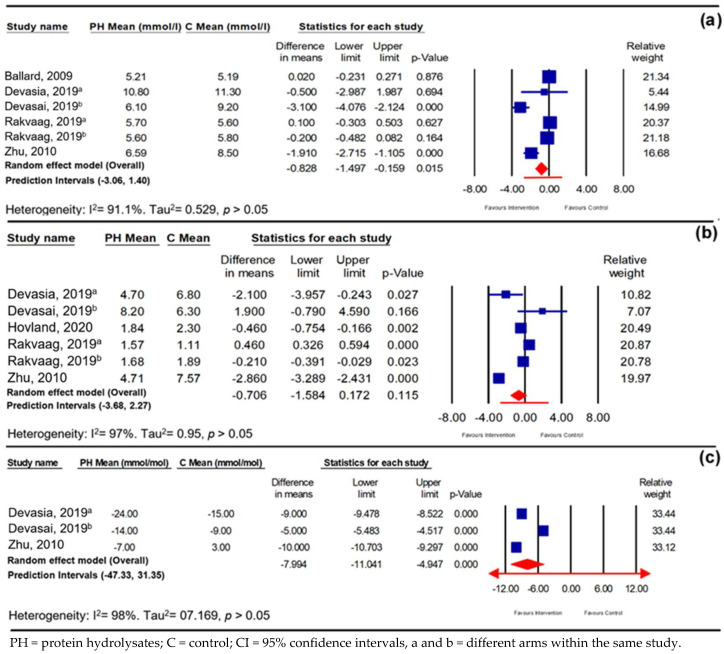
Pooled effect using inverse-variance random-effect model (mean difference and 95% CI) of long-term trials investigating effect of protein-hydrolysate intervention on fasting blood glucose (FBG) [41,43,53,55] (**a**), fasting glycated haemoglobin (HbA1c) [42,43,53,55] (**b**), and homeostatic model assessment of insulin resistance (HOMA-IR) [53,55] (**c**).

The meta-analysis revealed that there was a small but significant reduction in both fasting blood glucose (FBG) and HbA1c levels in response to PHs compared with the control group (*n* = 6; −0.83 mmol/L; 95% CI −1.50, −0.16; *p* ≤ 0.05, *I*^2^ = 91%; PI −3.06, 1.40, and −7.99 mmol/mol; 95% CI −11.04, −4.95; *p* ≤ 0.001, *I*^2^ = 98%; PI −47, 31.35) respectively (Figure 7a,b). Furthermore, PH supplementation was also able to reduce HOMA-IR in these human adults. However, this reduction was not significant when compared to the control group (*p* > 0.05) (Figure 7c). The subgroup analysis of long-term studies investigating the impact of the glycaemic status of the participants on the FBG in response to PH consumption reduced heterogeneity and showed that a greater reduction in FPG in the hyperglycaemic group (−2.10 mmol/L; 95% CI −3.24, −0.96; *p* ≤ 0.001, *I*^2^ = 55^%^) compared to the normoglycemic group (−0.05 mmol/L; 95% CI −0.22, 0.12; *p* > 0.05, *I*^2^ = 0%) could be achieved (Figure S17).

The GRADE assessment for each outcome, summarised in Table S3, revealed ‘moderate’ grades for acute PPGR in both the normoglycaemic and the hyperglycaemic adults, which were mainly downgraded due to the inconsistency and indirectness of these outcomes. The evidence for the long-term parameters of fasting glucose, HbA1c, was graded as ‘very low’ due to low ratings for consistency, directness, and precision, which led to a decrease in the level of certainty.

## 4. Discussion

The current systematic review and meta-analysis demonstrate that PH intake has glycaemia-regulatory effects in both acute postprandial responses and long-term indices. These findings are in line with previous in vitro studies suggesting possible mechanisms by which protein-hydrolysate-containing bioactive peptides could inhibit enzymes involved in glucose homeostasis. These peptides demonstrate a multifaceted impact on glycaemia by interfering with glucose absorption, mimicking insulin, enhancing insulin sensitivity, and inhibiting gluconeogenesis enzymes. Beyond these direct effects, BPs may also play a role in incretin-hormone modulation, enhancing overall glucose control. The inhibition of DPP-IV prolongs the activity of incretin hormones, while the modulation of glucose transporters facilitates efficient glucose uptake by cells. In addition, BPs inhibit carbohydrate digestion through enzymes like α-amylase and α-glucosidase, slowing down the breakdown of complex carbohydrates and reducing postprandial glucose levels [56,57]. For example, the potent inhibition of PHs against the α-glucosidase enzyme was reported, with an IC_50_ value of 0.0025 mg/mL, which is likely to lead to a marked reduction in the glucose available for absorption through the gastrointestinal tract [58].

Overall, the findings of the acute studies, comprising a total of over 550 subjects, showed some consistency in that the attenuation of postprandial glucose concentrations after the ingestion of an oral dose of PH was observed, with a mean reduction of 0.5 mmol/L, alongside elevations in plasma insulin responses (18 mIU/L), and more pronounced effects in the adults with hyperglycaemia.

Postprandial glycaemic management is considered crucial in the prevention of chronic diseases such as cardiovascular disease, in both normoglycaemic and T2DM individuals [59]. The estimated magnitude of the reduction in PPGR is similar to the reported effect of some glucose-lowering therapies, such as DPP-IV inhibitors [60]. However, different patterns in glucose and insulin response after a dose of PH were reported between studies and contributed to uncertainty in the prediction of future study effects. This variation in response could be due to a number of different factors, such as study design, participant age, BMI and health profile, and differences in physical activity and habitual diet, as well as the type of protein source or control, which might influence both the absorption of the PHs and their mechanisms of action. These potential cofounders were identified in acute RCTs and explored by subgroup, sensitivity, and meta-regression analyses.

It should be taken into account that differential patterns in insulin and glucose response after a dose of PH were reported between some studies. A dose-dependent relationship between the ingested amount of protein and the resulting glucose levels was previously reported in healthy subjects [29], which is in line with the present data, in which a dose-dependent response was observed between ≤10 g and 30 g, indicating that a small amount of BP had no [17] or only a small effect on postprandial glucose levels [22]. However, a study in which a very high dose (45 g) of BP was administered did not report any significant changes in terms of postprandial glucose levels [39]. These findings were confirmed by a meta-regression analysis of the dose–response relationship in acute RCTs, which revealed that consuming an amount of PH of around 30 g had the largest impact on PPGR in people with normoglycaemic profiles. This observation supports previous studies, which identified that Leucine is maximised at a protein dose of around 25 g, and that consuming 30+ g of protein in one sitting does not provide an additional boost [61]. Moreover, upon scrutinizing the study characteristics, it became apparent that the subgroup with high doses of PH were compared against whey isolate, while the does < 30 g were primarily compared to casein. Thus, the difference in effect could potentially be attributed to the different control groups, since whey protein is usually considered a rapidly digested protein compared to casein. Furthermore, the efficacy of an intervention may depend on the type/form of protein, i.e., its previous processing, which may determine the type, magnitude, and speed of BP generated, all of which influence its effects. For instance, in peptides extracted from native whey more proteins would remain intact, resulting in slower digestion, as well as lower plasma concentrations of amino acids, compared to the more common whey protein concentrate from cheese production [60]. This might explain the lack of effect of insulin treatment in a previous study [16], in which whey hydrolysate derived from a native protein source was used, albeit at a relatively low dose of 1.4 g, which might be excessively low to exert a significant response.

The ingestion of PH, as opposed to its intact protein, is assumed to involve accelerated protein digestion and absorption from the gut, with a stronger impact on postprandial glucose regulation [35]. In addition, recent in vitro research using preadipocyte tissue revealed that the low molecular weights of protein fractions among all protein concentrates was responsible for its potent biological activity, which was made evident through faster digestion and the availability of insulinotropic amino acids in the blood [62].

Interestingly, studies in which the same type of PH was used (whey protein) reported opposing results with regards to postprandial insulin secretion. While some studies [39] found a significant increase in postprandial insulin levels, others failed to show a similar treatment effect [16]. A small but not significant change was seen in a chronic trial in which an intervention of whey peptide was administered for 6 weeks. As indicated previously, insulinotropic properties appear to originate from a specific postprandial plasma amino acid pattern with predominantly isoleucine, leucine, lysine, threonine, and valine shown to directly stimulate beta cells to secrete insulin [63].

In addition, the negative correlation between glycaemic and insulinotropic response that was observed is supported by previous in vitro data, suggesting that glucose response to hydrolysed protein does not necessarily affect insulin release, and vice versa [64]. Therefore, one might assume that the reduction in glucose levels reported in some studies might have resulted from an insulin-independent pathway.

With regard to the long-term treatment effect of BP on blood glucose metabolism, despite the small number of chronic studies found, an overall treatment effect was found to be beneficial across the four studies, especially for fasting blood glucose and HbA1c. In contrast, the meta-analysis of the HOMA-IR data revealed a small, but not significant change in insulin resistance following the PH intervention. This finding can be explained by the small number of long-term studies included in the analysis and the variations in the study duration. Indeed, the strongest effects of PH intervention that have been reported were in studies with large sample sizes and of longer durations [53,55]. However, there is some evidence available related to HOMA-IR: the tripeptides IPP (Ile-Pro-Pro) and VPP (Val-Pro-Pro), derived from milk PHs, improved insulin sensitivity in diet-induced obese mice by decreasing pro-inflammatory cytokines in adipose tissue [65].

Bioactive peptides from marine sources have been particularly highlighted for their potency. For example, fish-collagen-derived peptides have been used in a number of studies. Overall, this suggests that marine-derived peptides have potential as supplements for T2DM patients to improve insulin sensitivity and glucose metabolism. Indeed, in vitro data suggested that the Gly-Pro-Hyp in fish-collagen hydrolysates is the main BP responsible for DPP-IV inhibition [66]. However, a recent 8-week intervention study on overweight individuals (average BMI 32.5 ± 3 kg/m^2^) did not demonstrate improvements in fasting glucose or insulin levels. Beyond the small sample size and relatively low dose, this lack of effect can be linked to the administration of fixed doses of 4 g of peptides rather than body-weight-adjusted doses, which would accommodate differences due to body-weight variations [67].

Regarding the strengths of this study, the current systematic review was performed to gather evidence from clinical RCTs. Although the study’s quality assessment using the Cochrane risk-of-bias tool suggested a ‘moderate risk’ of bias for numerous individual studies, their assessment as a pool of evidence using the GRADE tool suggested a generally ‘low’ or ‘very low’ risk of bias. There are some limitations to the present study, the most significant of which is the heterogeneity between the study data and how the authors reported their outcomes; however, this was mitigated through the sub-group analysis. Moreover, although RCTs were the main targeted studies for this review, only a small number of trials were found to have used placebo controls, with standard protein used as controls in other trials. Therefore, these were also explored to obtain a broader picture of the evidence available. Nevertheless, the uncertainty surrounding the bioavailability and absorption of PB is a crucial aspect in research. Factors like protein source, peptide/amino acid characteristics, and individual variations contribute to the complexity of this topic. Addressing these uncertainties and the main moderators of PHs’ effectiveness is vital for accurate interpretations and optimizing the potential health benefits of PHs and BPs.

## 5. Conclusions

To summarise, the current systematic review and meta-analysis confirmed the potential of PH to benefit postprandial glucose response, as demonstrated through the lowering of the postprandial glucose peak in normoglycaemic and in hyperglycaemic adults. Further, increasing age and existing hyperglycaemia were correlated with the increased efficacy of PH in lowering glucose. In contrast to the lowering of glucose, the acute studies demonstrated increased insulin levels overall, an effect that is likely to have been driven by different protein sources. The longer-term intake of protein hydrolysates lowered fasting glucose and fasting HbA1c, although further studies are required, on larger cohorts, to confirm the current findings. Overall, the PHs demonstrated their potential to improve glycaemia and should be considered in the prevention and management of hyperglycaemia and diabetes.

## Figures and Tables

**Table 1:** Summary of acute and long-term RCTs investigating the effects of protein hydrolysates on glycaemic markers in normoglycaemic individuals.

Study	Country	Sample Size (F, M)	Age (y) ^a^	BMI (kg/m^2^) ^a^	Design	Duration	Control	Protein Hydrolysates	Dose (g)
**Acute RCTs**									
Agergaard et al., 2021 [21]	Denmark	12 (6, 6)	69.0 ± 1	24.6 ± 1	C, SB	1 V	Steak protein	Meat	40
Akhavan et al., 2010 [22]	Canada	22 (10, 12)	25.1 ± 2	23.4 ± 3	C, DB	1 V	Placebo (CHO)	Whey	10
Ballard et al., 2013 [23]	USA	21 (10, 11)	55.0 ± 6	27.8 ± 2	C, DB	1 V	Placebo (CHO)	Whey	5
Bendtsen et al., 2014 [24]	Denmark	24 (5, 19)	29.0 ± 6	30.1 ± 2	C, DB	1 V	Casein isolate	Casein	30
Calbet et al., 2002 [25]	Denmark	6 (3/3)	22.7 ± 2	23.3 ± 6	C, DB	1 V	Protein isolate	Pea and whey	18
Chen et al., 2020 [26]	UK	20 (6/14)	26.0 ± 7	23.7 ± 2	C, DB	1 V	Milk	Whey	50
Claassens et al., 2009 [27]	The Netherlands	8 (0, 8)	32.0 ± 13	23.7 ± 1	L, SB	1 V	Placebo (CHO)	Pea, rice, soy, gluten, whey, and egg	15
Claessens et al., 2007 [28]	The Netherlands	8 (0, 8)	28.5 ± 10	23.3 ± 2	C, SB	1 V	Placebo (CHO)	Whey	23
Claessens et al., 2008 [29]	The Netherlands	12 (0, 12)	23.8 ± 3	23.0 ± 2	L, SB	1 V	Whey and soy isolate	Whey and soy	31 (22, 28 & 43)
Curran et al., 2019 [30]	Ireland	20 (9, 11)	50.0 ± 8	30.2 ± 3	C, DB	1 V	Casein isolate	Casein	12
Dale et al., 2018 [17]	Norway	41 (26, 15)	53.0 ± 1	25.0 ± 1	C, DB	1 V	Casein isolate	Marine	1.6
Deglaire et al., 2009 [31]	France	21 (12, 9)	30.0 ± 9	22.9 ± 4	P, DB	1 V	Casein isolate	Casein	NR
Drummond et al., 2018 [32]	Ireland	62 (NR)	53.6 ± 7	31.3 ± 5	C, DB	1 V	Casein isolate	Casein	12
Holmer-Jensen et al., 2012 [33]	Denmark	11 (6, 5)	60.4 ± 10	35.3 ± 4	C, SB	1 V	Whey isolate	Whey	45
Horner et al., 2019 [34]	Ireland	9 (3, 6)	59.5 ± 7	28.4 ± 3	C, DB	1 V	Casein isolate	Casein	12
Koopman et al., 2009 [35]	The Netherlands	10 (0, 10)	64.0 ± 3	24.7 ± 2	C, DB	1 V	Casein isolate	Casein	35
Manders et al., 2005 * [36]	The Netherlands	9 (0, 9)	58.2 ±1	27.5 ± 1	C, DB	1 V	Placebo (CHO)	Casein	30
Manders et al., 2006 * [37]	The Netherlands	10 (0, 10)	60.2 ± 1	27.2 ± 1	C, DB	1 V	Placebo (CHO)	Casein	25
Nakayama et al., 2018 [38]	Japan	11 (0, 11)	24.5 ±1	20.5 ± 1	C, DB	1 V	EAA mixture	Whey	5 (3.3, 5 & 7.5)
Power et al., 2009 [39]	Ireland	16 (0, 16)	22.4 ± 1	23.2 ± 1	C, DB	1 V	Whey isolate	Whey	45
Van-Loon et al., 2000 [40]	The Netherlands	8 (0, 8)	21.0 ± 1	21.4 ± 2	C, DB	1 V	Placebo and casein isolate	Pea, whey, and wheat	30
**Long-term RCTs**									
Ballard et al., 2009 [41]	USA	20 (10, 10)	25± 5	24.3 ± 2	C, DB	Daily-2 w	Placebo (CHO)	Whey	5
Hovland et al., 2020 [42]	Germany	49 (32, 27)	40.9 ± 2	33.2 ± 3	P, DB	Daily-8 w	Casein/whey mixture	Herring and salmon	2.5
Rakvaag et al., 2019 * [43]	Denmark	66 (34, 31)	58–67	29.4–30.3	P, DB	Daily-12 w	Starch	Whey	60

^a^ = data are expressed as means ± SEM, unless otherwise specified; C = crossover study design; DB = double blind; SB = single blind; L = Latin square; P = parallel; F = female; M = male; CHO = Carbohydrates; * = a study with multiple arms, each having its own distinct control group; BMI = Body mass index; NR = not reported; V = number of visits; w = weeks; EAA = essential amino acids.

**Table 2:** Summary of acute and long-term RCTs investigating the effect of protein hydrolysates on glycaemic markers in hyperglycaemic individuals.

Study	Country	Sample Size (F, M)	Age (y) ^a^	BMI (kg/m^2^) ^a^	Design	Duration	Control	Protein Hydrolysates	Dose (g)
**Acute RCTs**									
Geerts et al., 2011 [44]	The Netherlands	36 (9, 27)	61.5 ± 5	28.1 ± 4	PC, DB	1 V	Placebo (CHO)	Casein	15
Goudarzi et al., 2013 [45]	Iran	10 (0, 10)	32.4 ± 4	26.2 ± 1	C, DB	1 V	Placebo & whey isolate	Whey	18 (8, 16 & 32)
Hoefle et al., 2018 [46]	Germany	15 (5, 10)	62.0 ± 7	29.0 ± 6	C, SB	1 V	Placebo & whey isolate	Glycomacropeptides	50
Jonker et al., 2011 [47]	The Netherlands	13 (5, 8)	58.0 ± 1	27.9 ± 1	C, DB	1 V	Placebo (CHO)	Casein	12
King et al., 2018 [48]	UK	11 (0, 11)	54.9 ± 2	31.8 ± 3	C, SB	1 V	Placebo (CHO)	Whey	15
Manders et al., 2005 * [36]	The Netherlands	10 (0, 10)	61.5 ± 2	27.2 ± 1	C, DB	1 V	Placebo (CHO)	Casein	29
Manders et al., 2006 * [37]	The Netherlands	10 (0, 10)	59.7 ± 3	26.8 ± 1	C, DB	1 V	Placebo (CHO)	Casein	25
Manders et al., 2009 [49]	The Netherlands	13 (0, 13)	62.0 ± 2	28.0 ± 1	C, DB	1 V	Placebo (CHO)	Casein	26
Manders et al., 2014 [50]	The Netherlands	60 (0, 60)	60.0 ± 1	30.2 ± 1	C, DB	1 V	Placebo (CHO)	Casein	28
Mortensen et al., 2012 [51]	Denmark	12 (7, 5)	65.8 ± 5	28.2 ± 5	C, SB	1 V	Whey isolate	Whey	45
Plat et al., 2019 [52]	The Netherlands	40 (30, 10)	18–70	25–35	C, DB	1 V	Maize Starch	Egg	5
Sartorius et al., 2019 [16]	Germany	21 (13, 8)	62.4 ± 3	28.1 ± 2	C, DB	1 V	Placebo (CHO)	Whey	2 (1.6 & 2.4)
**Long-term RCTs**									
Devasia et al., 2019 * [53]	India	60 (33, 27)	21.0 ± 1	27.4 ± 1	P, DB	Daily-12 w	Starch	Marine, collagen	2.5
Jensen et al., 2020 [54]	Norway	20 (21, 9)	53.0 ± 6	32.5 ± 3	P, DB	Daily-8 w	Placebo (CHO)	Cod	4
Sartorius et al., 2019 [16]	Germany	21 (13, 8)	62.4 ± 3	28.1 ± 2	SA	Daily-6 w	No control	Whey	1.4
Zhu et al., 2010 [55]	China	100 (49, 51)	63.3 ± 1	24.2 ± 1	P, DB	Daily-12 w	Carboxymethylcellulose	Marine, collagen	16.5

^a^ = data are expressed as means ± SEM, unless otherwise specified; C = crossover study design; PC = partially crossover study design; DB = double blind; P = parallel; SA = single-arm; F = female; M = male; CHO = carbohydrates; * = a study with multiple arms, each having its own distinct control group; BMI = body mass index; V = number of visits; w = weeks.

## Data Availability

Not applicable.

## Supplementary Materials

The following supporting information can be downloaded at: https://www.mdpi.com/article/10.3390/nu16020323/s1, Table S1: Database-search terms; Table S2: Risk-of-bias assessment; Table S3: GRADE assessment; Figure S1: Subgroup analysis of acute studies investigating the effect of different doses of protein-hydrolysate consumption on postprandial glucose response in adults with normoglycaemia; Figure S2: Subgroup analysis by control type on effects of acute studies investigating acute glucose response after protein-hydrolysate consumption in adults with normoglycaemia; Figure S3: Subgroup analysis by BMI on acute studies investigating postprandial glucose response after protein-hydrolysate consumption in normoglycaemic adults; Figure S4: Subgroup analysis of acute studies investigating the impact of the participants’ age on the postprandial glucose in response to protein-hydrolysate consumption in adults with normoglycaemia; Figure S5: Subgroup analysis of acute studies investigating the effects of different doses of protein-hydrolysate consumption on postprandial glucose response in adults with hyperglycaemia; Figure S6: Sensitivity analysis conducted by removing one study based on BMI from among the studies investigating postprandial glucose response after protein-hydrolysate consumption in adults with hyperglycaemia; Figure S7: Sensitivity analysis by removing two studies based on control type of the studies investigating glucose response after protein hydrolysates consumption in adults with hyperglycaemia; Figure S8: Sensitivity analysis conducted by removing one study based on participants’ age from among the studies investigating postprandial glucose response after protein-hydrolysate consumption in adults with hyperglycaemia; Figure S9: Subgroup analysis by control type on acute studies investigating postprandial insulin response after protein-hydrolysate consumption in normoglycaemic adults; Figure S10: Subgroup analysis by BMI on acute studies investigating postprandial insulin response after protein-hydrolysate consumption in adults with normoglycaemia; Figure S11: Subgroup analysis of acute studies investigating the effects of different doses of protein-hydrolysate consumption on postprandial insulin response in adults with normoglycaemia; Figure S12: Subgroup analysis of acute studies investigating the impact of the participants’ age on the postprandial insulin in response to protein-hydrolysate consumption in adults with normoglycaemia; Figure S13: Subgroup analysis of acute studies investigating the effects of different doses of protein-hydrolysate consumption on postprandial insulin response in adults with hyperglycaemia; Figure S14: Sensitivity analysis conducted by removing two studies based on control type from among the studies investigating postprandial insulin response after protein-hydrolysate consumption in adults with hyperglycaemia; Figure S15: Sensitivity analysis conducted by removing one study based on BMI from among the studies investigating postprandial insulin response after protein-hydrolysate consumption in adults with hyperglycaemia; Figure S16: Sensitivity analysis conducted by removing one study based on participants’ age from among the studies investigating postprandial insulin response after protein-hydrolysate consumption in adults with hyperglycaemia; Figure S17: Subgroup analysis of long-term studies investigating the impact of the glycaemic status of the participants on fasting glucose levels in response to protein-hydrolysate consumption.
