# Supplementary material for: Protein Hydrolysates and Bioactive Peptides as Mediators of Blood Glucose—A Systematic Review and Meta-Analysis of Acute and Long-Term Studies
Source: Nutrients. 2024 Jan 22;16(2):323. doi: 10.3390/nu16020323 (PMC10818427; doi:10.3390/nu16020323)
Supplement: Supplementary file 1 [file nutrients-16-00323-s001.zip › nutrients-2790326-supplementary.pdf]

Supplementary data manuscript Nutrients, Elbira et al.

Protein Hydrolysates and Bioactive Peptides as Mediators of Blood Glucose—A Systematic Review and Meta-Analysis of Acute and Long-Term Studies

Table S1. Database search terms.

Search Category	Search Terms Used
**1. Population**	(normoglycaemic) OR (normoglycaemia) OR (normoglycemia) OR (healthy) OR (normal) OR (diabetic) OR (diabetes) OR (Prediabetic) OR (prediabetes) OR (T2DM) OR (T2D) OR (NIDDM) OR (adults)
**2. Intervention**	(hydrolysates) OR (bioactive peptides) OR (peptides) OR (hydrolysed protein) OR (protein extracts)
**3. Control**	(placebo) OR (protein) OR (intact protein) OR (carbohydrates) OR (CHO) OR (casein) OR (whey) OR (protein isolate) OR (protein concentrate)
**4. Outcomes**	(glucose) OR (FPG) OR (PPG) OR (glycemic) OR (glycaemia) OR (glycemia) OR (insulin) OR (post-prandial) OR (postprandial) OR (PPGR) OR (OGTT) OR (insulinemic) OR (insulinaemic) OR (HOMA) OR (Homeostatic Model Assessment of Insulin Resistance) OR insulin resistance) OR (IR) OR (glycated hemoglobin) OR (HbA1c) OR (A1C) OR (A1c) OR (glycated haemoglobin) OR (glycosylated haemoglobin)

Table S2. Risk of bias assessment.

Study	Risk of Bias Assessment					
	Domain					
	Randomization Process	Deviation from Intended Intervention	Missing Outcome Data	Measurement of the Outcome	Selection of the Reported Results	Overall
**Akhavan et al., 2010**	some concerns	low risk	low risk	low risk	some concerns	some concerns
**Ballard et al., 2009**	some concerns	low risk	low risk	low risk	some concerns	some concerns
**Ballard et al., 2013**	low risk	low risk	some concerns	low risk	low risk	some concerns
**Bendtsen et al., 2014**	some concerns	low risk	some concerns	low risk	some concerns	some concerns
**Calbet et al., 2002**	some concerns	low risk	some concerns	low risk	some concerns	some concerns
**Chen, 2020**	some concerns	low risk	low risk	low risk	some concerns	some concerns
**Claessens, 2007**	some concerns	Some concerns	some concerns	low risk	some concerns	high risk
**Claessens, 2008**	some concerns	Some concerns	low risk	low risk	some concerns	some concerns
**Claessens, 2009**	some concerns	Some concerns	low risk	low risk	low risk	some concerns
**Curran, 2019**	some concerns	low risk	low risk	low risk	some concerns	some concerns
**Dale, 2018**	low risk	low risk	low risk	low risk	low risk	low risk
**Deglaire, 2009**	low risk	low risk	low risk	low risk	some concerns	some concerns
**Devasia, 2019**	some concerns	low risk	some concerns	low risk	some concerns	high risk
**Drummond, 2018**	some concerns	low risk	low risk	low risk	some concerns	some concerns
**Geerts, 2011**	some concerns	low risk	low risk	low risk	some concerns	some concerns
**Goudarzi, 2013**	some concerns	low risk	some concerns	low risk	some concerns	high risk
**Hoefle, 2019**	low risk	low risk	low risk	low risk	some concerns	some concerns

**Holmer-Jensen, 2012**	some concerns	low risk	low risk	low risk	some concerns	some concerns
**Horner, 2019**	low risk	low risk	low risk	low risk	some concerns	some concerns
**Hovaland, 2020**	low risk	low risk	low risk	low risk	some concerns	some concerns
**Jensen, 2020**	some concerns	low risk	low risk	low risk	some concerns	some concerns
**Jonker, 2011**	some concerns	low risk	low risk	low risk	some concerns	some concerns
**King, 2018**	some concerns	low risk	some concerns	low risk	some concerns	high risk
**Koopman, 2009**	some concerns	low risk	some concerns	low risk	some concerns	high risk
**Mandres, 2005**	some concerns	low risk	Some concerns	low risk	some concerns	high risk
**Mandres, 2006**	low risk	low risk	low risk	low risk	some concerns	some concerns
**Mandres, 2009**	some concerns	low risk	low risk	low risk	some concerns	some concerns
**Mandres, 2014**	some concerns	low risk	low risk	low risk	some concerns	some concerns
**Mortensen, 2012**	some concerns	low risk	low risk	low risk	some concerns	some concerns
**Nakayama, 2018**	some concerns	low risk	low risk	low risk	some concerns	some concerns
**Plat, 2019**	low risk	low risk	low risk	low risk	some concerns	some concerns
**Power, 2009**	low risk	low risk	low risk	low risk	some concerns	some concerns
**Rakvaag, 2019**	some concerns	low risk	low risk	low risk	some concerns	some concerns
**Sartorius, 2019**	low risk	low risk	low risk	low risk	some concerns	some concerns
**Van-Loon, 2000**	some concerns	low risk	some concerns	low risk	some concerns	high risk
**Zhu, 2010**	some concerns	low risk	low risk	low risk	some concerns	some concerns

Table S3. GRADE assessment.

Outcome	No. of Trials	No. of Participants	Certainty assessment					Effect Estimate	Grade
			Risk of Bias	Inconsistency	Indirectness	Imprecision	Other Considerations		
**PPGR normoglycemia**	15	573	Not serious	Not serious	Serious [b]	Not serious	None	−0.22 [−0.40, −0.01]	Moderate
**PPGR hyperglycaemia**	8	342	Not serious	Not serious	Serious [b]	Not serious	None	−0.88 [−1.37, −0.39]	Moderate
**PPIR normoglycaemia**	12	470	Not serious	Serious [a]	Serious [b]	Not serious	None	12.98 [2.73, 23.23]	Low
**PPIR hyperglycaemia**	7	322	Not serious	Serious [a]	Serious [b]	Not serious	None	23.05 [7.53, 38.57]	Low
**FBG**	6	266	Not serious	Serious [a]	Serious [b]	Serious [c]	None	−0.83 [−1.50, −0.16]	Very Low
**HbA_{1c}**	3	161	Not serious	Serious [a]	Serious [b]	Very serious [e]	None	−7.99 [−11.04, −4.95]	Very Low
**HOMA-IR**	6	306	Not serious	Serious [a]	Serious [b]	Very serious [c,d]	None	−0.71 [−1.58, 0.17]	Very low

PPGR, Postprandial Glucose Response; PPIR, Postprandial Insulin Response; FBG, Fasting Blood Glucose; HbA_{1c}, Glycated haemoglobin; HOMA-IR, homeostatic model assessment of insulin resistance; a. Due to unexplained variability among the studies; b. Due to variations in the control groups; c. Different arms with same publications or different studies with same authors. d. The 95% confidence interval encompassed both positive effects and adverse effects. e. Due to small number of studies.

a. High heterogeneity between studies that was not improved through further subgroup analyses, meta-regression, or sensitivity analyses.

b. There was no standardized control for all studies. Control varied between carbohydrates, reference popular proteins such as Casein or Whey, isolate protein (unhydrolysed), milk and mixture of protein and amino acids. In addition, the form of the control was different in some studies; some were given as drinks others as tables.

c. Publications bias may occur when including different studies/arms with same authors as authors may consistently report similar findings across multiple studies, there may be a risk of confirmation bias. This occurs when researchers selectively publish or emphasize studies that confirm their hypotheses while neglecting those that do not.

d. No significant change. The change was in the same direction with the intervention, however the 95% confident intervals crossed one (-1.58 to 0.17).

e. only 3 studies were included in the analysis. With a small number of studies, it may be challenging to assess and explain heterogeneity. Even minor differences between studies can disproportionately influence the overall results, and there may not be enough statistical power to detect and understand sources of heterogeneity.

Study or Subgroup	Protein hydrolysates Mean [mmol/L]	SD [mmol/L]	Total	Control Mean [mmol/L]	SD [mmol/L]	Total	Weight	Mean Difference IV, Random, 95% CI
**1.1.1 Dose (≤10g)**								
Akhavan, 2010	7.2	0.69	21	7.4	0.9	21	9.4%	-0.20 [-0.69, 0.29]
Ballard, 2013	5	0.6	21	5.1	0.6	21	12.1%	-0.10 [-0.46, 0.26]
Dale, 2018	7.5	1.8	41	7.4	1.5	41	5.9%	0.10 [-0.62, 0.82]
**Subtotal (95% CI)**			**83**			**83**	**27.4%**	**-0.10 [-0.37, 0.17]**
Heterogeneity: Tau² = 0.00; Chi² = 0.46, df = 2 (P = 0.79); I² = 0%								
Test for overall effect: Z = 0.75 (P = 0.46)								
**1.1.2 Dose (11- 20g)**								
Calbet, 2002	5.6	0.73	6	6.1	0.63	6	5.3%	-0.50 [-1.27, 0.27]
Curran, 2018	7.7	1.55	20	8.4	1.83	20	3.3%	-0.70 [-1.75, 0.35]
Drummond, 2018	7.1	0.79	62	7.2	0.79	62	14.2%	-0.10 [-0.38, 0.18]
Horner, 2019	7.1	1.3	9	7.3	1.68	9	2.1%	-0.20 [-1.59, 1.19]
**Subtotal (95% CI)**			**97**			**97**	**24.9%**	**-0.18 [-0.43, 0.07]**
Heterogeneity: Tau² = 0.00; Chi² = 1.92, df = 3 (P = 0.59); I² = 0%								
Test for overall effect: Z = 1.40 (P = 0.16)								
**1.1.3 Dose (21-30g)**								
Bendtsen, 2014	6	1.3	24	6.6	1.2	24	6.0%	-0.60 [-1.31, 0.11]
Claessens, 2007	5.4	0.48	8	6.9	1.23	8	4.1%	-1.50 [-2.41, -0.59]
Manders, 2005¹	8.9	1.17	9	10.4	1.41	9	2.7%	-1.50 [-2.70, -0.30]
Manders, 2006¹	8.5	2.73	10	10.2	1.47	10	1.1%	-1.70 [-3.62, 0.22]
Van-Loon, 2000	6.6	1.08	8	6.9	0.98	8	3.5%	-0.30 [-1.31, 0.71]
**Subtotal (95% CI)**			**59**			**59**	**17.5%**	**-0.97 [-1.50, -0.44]**
Heterogeneity: Tau² = 0.09; Chi² = 5.31, df = 4 (P = 0.26); I² = 25%								
Test for overall effect: Z = 3.59 (P = 0.0003)								
**1.1.4 Dose (>30g)**								
Chen, 2020	4.9	0.95	20	4.7	0.52	21	9.7%	0.20 [-0.27, 0.67]
Holmer-Jensen, 2012	5.8	0.41	11	5.8	0.61	11	10.5%	0.00 [-0.43, 0.43]
Power, 2009	4.05	0.7	16	3.9	0.61	16	10.0%	0.15 [-0.30, 0.60]
**Subtotal (95% CI)**			**47**			**48**	**30.2%**	**0.11 [-0.15, 0.37]**
Heterogeneity: Tau² = 0.00; Chi² = 0.42, df = 2 (P = 0.81); I² = 0%								
Test for overall effect: Z = 0.83 (P = 0.41)								
**Total (95% CI)**			**286**			**287**	**100.0%**	**-0.23 [-0.44, -0.02]**
Heterogeneity: Tau² = 0.06; Chi² = 24.18, df = 14 (P = 0.04); I² = 42%								
Test for overall effect: Z = 2.11 (P = 0.03)								
Test for subgroup differences: Chi² = 13.12, df = 3 (P = 0.004), I² = 77.1%								

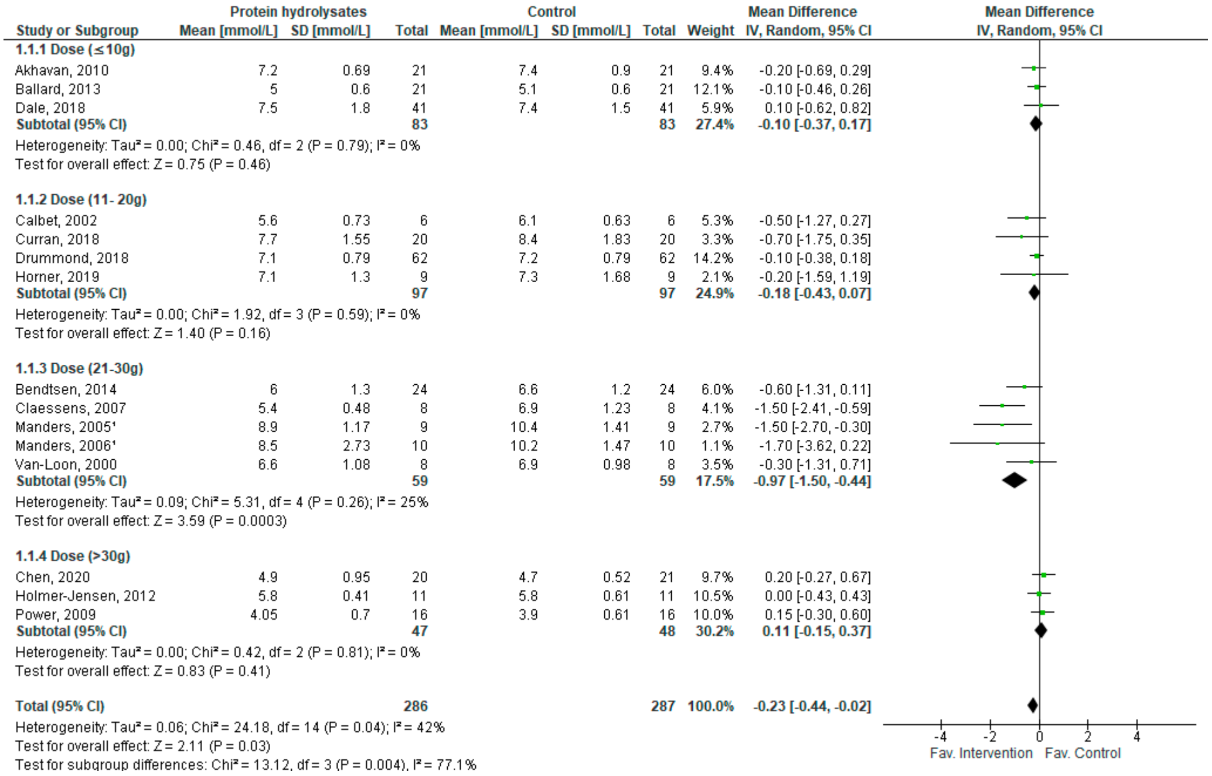

Figure S1. Subgroup analysis of acute studies investigating the effect of different doses of protein hydrolysate consumption on postprandial glucose response in adults with normoglycaemia.

Study or Subgroup	Protein hydrolysates Mean [mmol/L]	SD [mmol/L]	Total	Control Mean [mmol/L]	SD [mmol/L]	Total	Weight	Mean Difference IV, Random, 95% CI
**1.1.1 CHO**								
Akhavan, 2010	7.2	0.69	21	7.4	0.9	21	9.4%	-0.20 [-0.69, 0.29]
Ballard, 2013	5	0.6	21	5.1	0.6	21	11.7%	-0.10 [-0.46, 0.26]
Chen, 2020	4.9	0.95	20	4.7	0.52	21	9.6%	0.20 [-0.27, 0.67]
Claessens, 2007	5.4	0.48	8	6.9	1.23	8	4.4%	-1.50 [-2.41, -0.59]
Manders, 2005¹	8.9	1.17	9	10.4	1.41	9	2.9%	-1.50 [-2.70, -0.30]
Manders, 2006¹	8.5	2.73	10	10.2	1.47	10	1.3%	-1.70 [-3.62, 0.22]
**Subtotal (95% CI)**			**89**			**90**	**39.4%**	**-0.51 [-1.02, 0.01]**
Heterogeneity: Tau² = 0.25; Chi² = 17.66, df = 5 (P = 0.003); I² = 72%								
Test for overall effect: Z = 1.93 (P = 0.05)								
**1.1.2 Unhydrolysed protein**								
Bendtsen, 2014	6	0.91	24	6.6	0.83	24	9.3%	-0.60 [-1.09, -0.11]
Calbet, 2002	5.6	0.73	6	6.1	0.63	6	5.6%	-0.50 [-1.27, 0.27]
Curran, 2018	7.7	1.55	20	8.4	1.83	20	3.6%	-0.70 [-1.75, 0.35]
Dale, 2018	7.5	1.8	41	7.4	1.5	41	6.2%	0.10 [-0.62, 0.82]
Drummond, 2018	7.1	0.79	62	7.2	0.79	62	13.3%	-0.10 [-0.38, 0.18]
Holmer-Jensen, 2012	5.8	0.41	11	5.8	0.61	11	10.3%	0.00 [-0.43, 0.43]
Horner, 2019	7.1	1.3	9	7.3	1.68	9	2.3%	-0.20 [-1.59, 1.19]
Power, 2009	4.1	0.7	16	3.9	0.61	16	9.9%	0.20 [-0.25, 0.65]
**Subtotal (95% CI)**			**189**			**189**	**60.6%**	**-0.14 [-0.35, 0.07]**
Heterogeneity: Tau² = 0.01; Chi² = 8.32, df = 7 (P = 0.30); I² = 16%								
Test for overall effect: Z = 1.34 (P = 0.18)								
**Total (95% CI)**			**278**			**279**	**100.0%**	**-0.24 [-0.47, -0.02]**
Heterogeneity: Tau² = 0.08; Chi² = 26.32, df = 13 (P = 0.02); I² = 51%								
Test for overall effect: Z = 2.13 (P = 0.03)								
Test for subgroup differences: Chi² = 1.68, df = 1 (P = 0.20), I² = 40.4%								

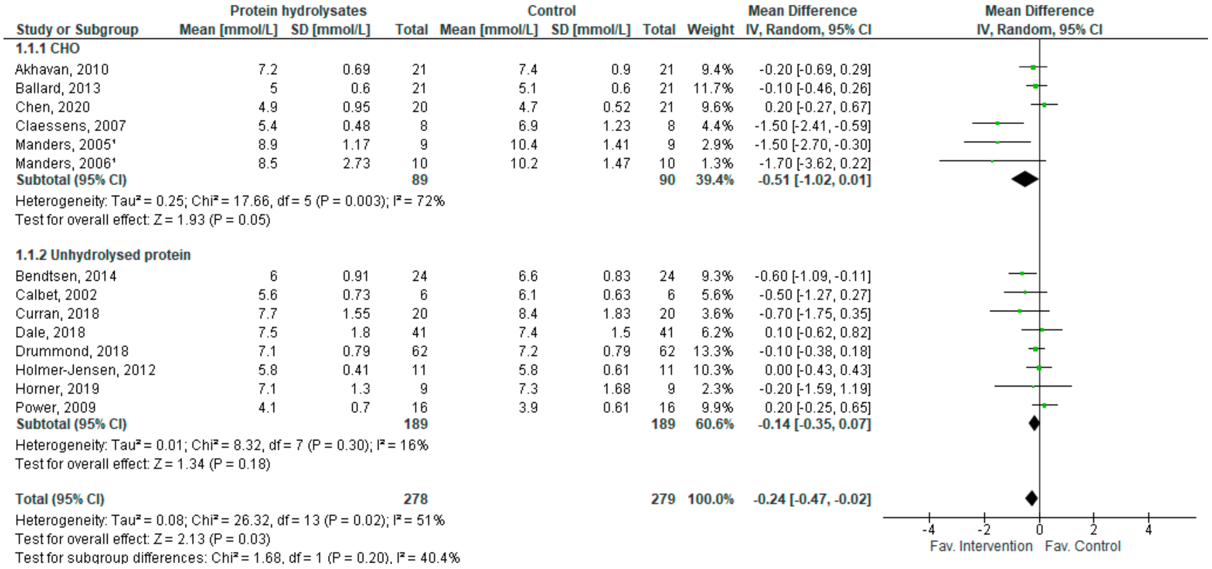

Figure S2. Subgroup analysis by control type on acute studies investigating acute glucose response after protein hydrolysates consumption in adults with normoglycaemia.

Study or Subgroup	Protein hydrolysates Mean [mmol/L]	SD [mmol/L]	Total	Control Mean [mmol/L]	SD [mmol/L]	Total	Weight	Mean Difference IV, Random, 95% CI
**1.1.1 BMI≤ 25 kg/m^2**								
Akhavan, 2010	7.2	0.69	21	7.4	0.9	21	9.1%	-0.20 [-0.69, 0.29]
Calbet, 2002	5.6	0.73	6	6.1	0.63	6	5.3%	-0.50 [-1.27, 0.27]
Chen, 2020	4.9	0.95	20	4.7	0.52	21	9.3%	0.20 [-0.27, 0.67]
Claessens, 2007	5.4	0.48	8	6.9	1.23	8	4.2%	-1.50 [-2.41, -0.59]
Dale, 2018	7.5	1.8	41	7.4	1.5	41	5.9%	0.10 [-0.62, 0.82]
Power, 2009	4.1	0.7	16	3.9	0.61	16	9.6%	0.20 [-0.25, 0.65]
Van-Loon, 2000	6.6	1.08	8	6.9	0.98	8	3.6%	-0.30 [-1.31, 0.71]
**Subtotal (95% CI)**			**120**			**121**	**47.0%**	**-0.19 [-0.55, 0.18]**
Heterogeneity: Tau² = 0.13; Chi² = 13.85, df = 6 (P = 0.03); I² = 57%								
Test for overall effect: Z = 1.00 (P = 0.32)								
**1.1.2 BMI> 25 kg/m^2**								
Ballard, 2013	5	0.6	21	5.1	0.6	21	11.4%	-0.10 [-0.46, 0.26]
Bendtsen, 2014	6	0.91	24	6.6	0.83	24	9.0%	-0.60 [-1.09, -0.11]
Curran, 2018	7.7	1.55	20	8.4	1.83	20	3.4%	-0.70 [-1.75, 0.35]
Drummond, 2018	7.1	0.79	62	7.2	0.79	62	13.2%	-0.10 [-0.38, 0.18]
Holmer-Jensen, 2012	5.8	0.41	11	5.8	0.61	11	10.0%	0.00 [-0.43, 0.43]
Horner, 2019	7.1	1.3	9	7.3	1.68	9	2.1%	-0.20 [-1.59, 1.19]
Manders, 2005¹	8.9	1.17	9	10.4	1.41	9	2.7%	-1.50 [-2.70, -0.30]
Manders, 2006¹	8.5	2.73	10	10.2	1.47	10	1.2%	-1.70 [-3.62, 0.22]
**Subtotal (95% CI)**			**166**			**166**	**53.0%**	**-0.30 [-0.57, -0.02]**
Heterogeneity: Tau² = 0.05; Chi² = 11.86, df = 7 (P = 0.11); I² = 41%								
Test for overall effect: Z = 2.13 (P = 0.03)								
**Total (95% CI)**			**286**			**287**	**100.0%**	**-0.24 [-0.46, -0.03]**
Heterogeneity: Tau² = 0.07; Chi² = 26.39, df = 14 (P = 0.02); I² = 47%								
Test for overall effect: Z = 2.20 (P = 0.03)								
Test for subgroup differences: Chi² = 0.22, df = 1 (P = 0.64), I² = 0%								

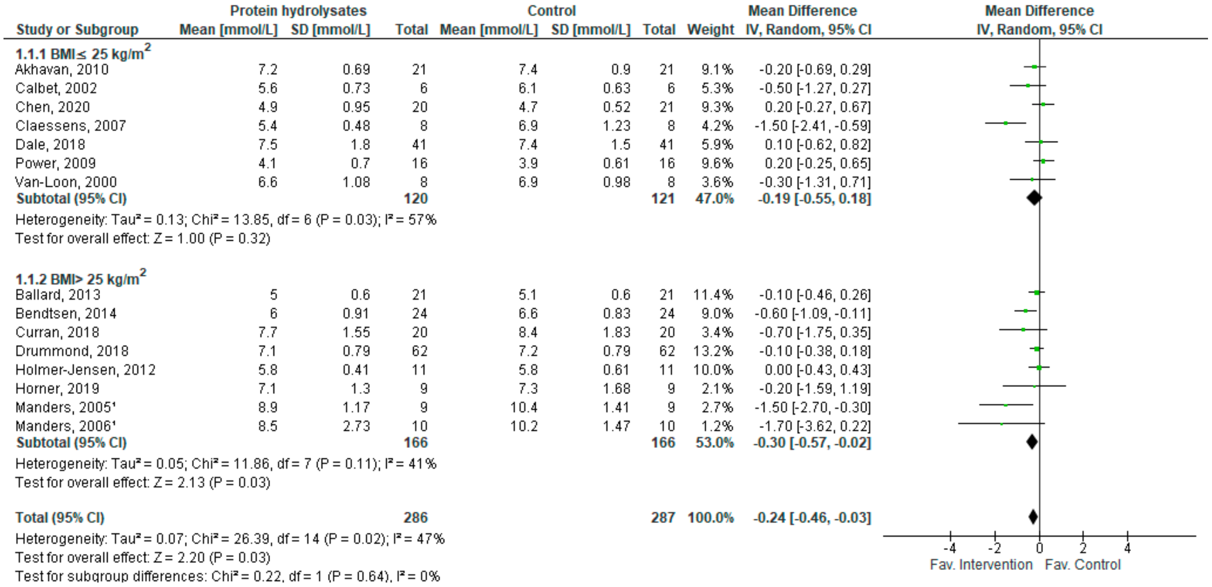

Figure S3. Subgroup analysis by BMI on acute studies investigating postprandial glucose response after protein hydrolysates consumption in normoglycaemic adults.

Study or Subgroup	Protein hydrolysates Mean [mmol/L]	SD [mmol/L]	Total	Control Mean [mmol/L]	SD [mmol/L]	Total	Weight	Mean Difference IV, Random, 95% CI
**1.1.1 18 to 35 y**								
Akhavan, 2010	7.2	0.69	21	7.4	0.9	21	9.1%	-0.20 [-0.69, 0.29]
Bendtsen, 2014	6	0.91	24	6.6	0.83	24	9.0%	-0.60 [-1.09, -0.11]
Calbet, 2002	5.6	0.73	6	6.1	0.63	6	5.3%	-0.50 [-1.27, 0.27]
Chen, 2020	4.9	0.95	20	4.7	0.52	21	9.3%	0.20 [-0.27, 0.67]
Claessens, 2007	5.4	0.48	8	6.9	1.23	8	4.2%	-1.50 [-2.41, -0.59]
Power, 2009	4.1	0.7	16	3.9	0.61	16	9.6%	0.20 [-0.25, 0.65]
Van-Loon, 2000	6.6	1.08	8	6.9	0.98	8	3.6%	-0.30 [-1.31, 0.71]
**Subtotal (95% CI)**			**103**			**104**	**50.1%**	**-0.30 [-0.68, 0.08]**
Heterogeneity: Tau² = 0.16; Chi² = 16.63, df = 6 (P = 0.01); I² = 64%								
Test for overall effect: Z = 1.56 (P = 0.12)								
**1.1.2 36 to 55 y**								
Ballard, 2013	5	0.6	21	5.1	0.6	21	11.4%	-0.10 [-0.46, 0.26]
Curran, 2018	7.7	1.55	20	8.4	1.83	20	3.4%	-0.70 [-1.75, 0.35]
Dale, 2018	7.5	1.8	41	7.4	1.5	41	5.9%	0.10 [-0.62, 0.82]
Drummond, 2018	7.1	0.79	62	7.2	0.79	62	13.2%	-0.10 [-0.38, 0.18]
**Subtotal (95% CI)**			**144**			**144**	**33.9%**	**-0.11 [-0.31, 0.10]**
Heterogeneity: Tau² = 0.00; Chi² = 1.55, df = 3 (P = 0.67); I² = 0%								
Test for overall effect: Z = 1.01 (P = 0.31)								
**1.1.3 56 y and older**								
Holmer-Jensen, 2012	5.8	0.41	11	5.8	0.61	11	10.0%	0.00 [-0.43, 0.43]
Horner, 2019	7.1	1.3	9	7.3	1.68	9	2.1%	-0.20 [-1.59, 1.19]
Manders, 2005¹	8.9	1.17	9	10.4	1.41	9	2.7%	-1.50 [-2.70, -0.30]
Manders, 2006¹	8.5	2.73	10	10.2	1.47	10	1.2%	-1.70 [-3.62, 0.22]
**Subtotal (95% CI)**			**39**			**39**	**16.0%**	**-0.66 [-1.55, 0.24]**
Heterogeneity: Tau² = 0.48; Chi² = 7.65, df = 3 (P = 0.05); I² = 61%								
Test for overall effect: Z = 1.44 (P = 0.15)								
**Total (95% CI)**			**286**			**287**	**100.0%**	**-0.24 [-0.46, -0.03]**
Heterogeneity: Tau² = 0.07; Chi² = 26.39, df = 14 (P = 0.02); I² = 47%								
Test for overall effect: Z = 2.20 (P = 0.03)								
Test for subgroup differences: Chi² = 1.96, df = 2 (P = 0.37), I² = 0%								

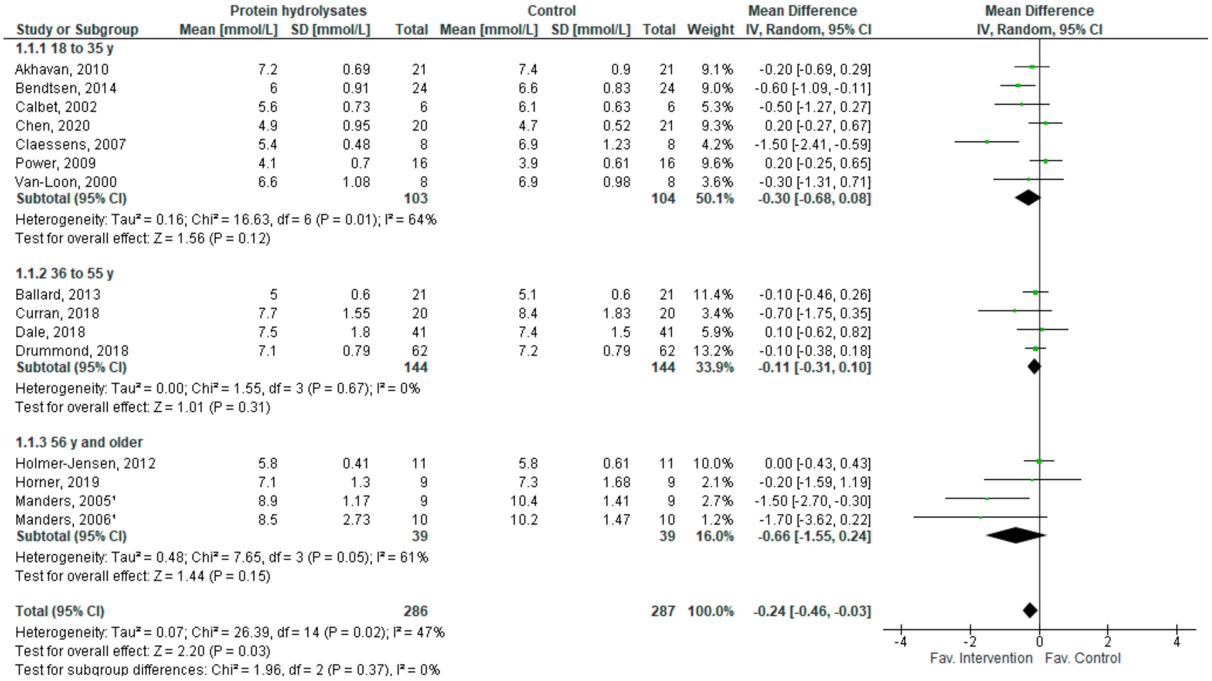

Figure S4. Subgroup analysis of acute studies investigating the impact of the participants' age on the postprandial glucose in response to protein hydrolysates consumption in adults with normoglycaemia.

Study or Subgroup	protein hydrolysates Mean [mmol/L]	SD [mmol/L]	Total	Control Mean [mmol/L]	SD [mmol/L]	Total	Weight	Mean Difference IV, Random, 95% CI
**1.1.1 Dose (≤20g)**								
Geerts, 2011	11.74	0.52	36	12.85	0.71	36	20.9%	-1.11 [-1.40, -0.82]
Goudarzi, 2013	11.17	1.6	10	12.7	1.75	10	7.6%	-1.53 [-3.00, -0.06]
Jonker, 2011	14.1	1.8	13	15	1.8	13	8.2%	-0.90 [-2.28, 0.48]
Sartorius, 2019	8.59	1.16	21	8.95	1.16	21	15.5%	-0.36 [-1.06, 0.34]
**Subtotal (95% CI)**			**80**			**80**	**52.3%**	**-0.93 [-1.37, -0.50]**
Heterogeneity: Tau² = 0.06; Chi² = 4.27, df = 3 (P = 0.23); I² = 30%								
Test for overall effect: Z = 4.19 (P < 0.0001)								
**1.1.2 Dose (>20g)**								
Manders, 2005ᵃ	15.58	1.99	10	16.6	2.35	10	5.2%	-1.02 [-2.93, 0.89]
Manders, 2006ᵃ	15.979	1.761	10	17.402	2.739	10	4.8%	-1.42 [-3.44, 0.60]
Manders, 2014	15.5	0.6	60	17	0.5	60	21.7%	-1.50 [-1.70, -1.30]
Mortensen, 2012	9.2	0.88	12	8.9	0.78	12	16.0%	0.30 [-0.37, 0.97]
**Subtotal (95% CI)**			**92**			**92**	**47.7%**	**-0.85 [-2.08, 0.38]**
Heterogeneity: Tau² = 1.17; Chi² = 25.96, df = 3 (P < 0.00001); I² = 88%								
Test for overall effect: Z = 1.35 (P = 0.18)								
**Total (95% CI)**			**172**			**172**	**100.0%**	**-0.88 [-1.38, -0.38]**
Heterogeneity: Tau² = 0.29; Chi² = 34.52, df = 7 (P < 0.0001); I² = 80%								
Test for overall effect: Z = 3.45 (P = 0.0006)								
Test for subgroup differences: Chi² = 0.02, df = 1 (P = 0.90), I² = 0%								

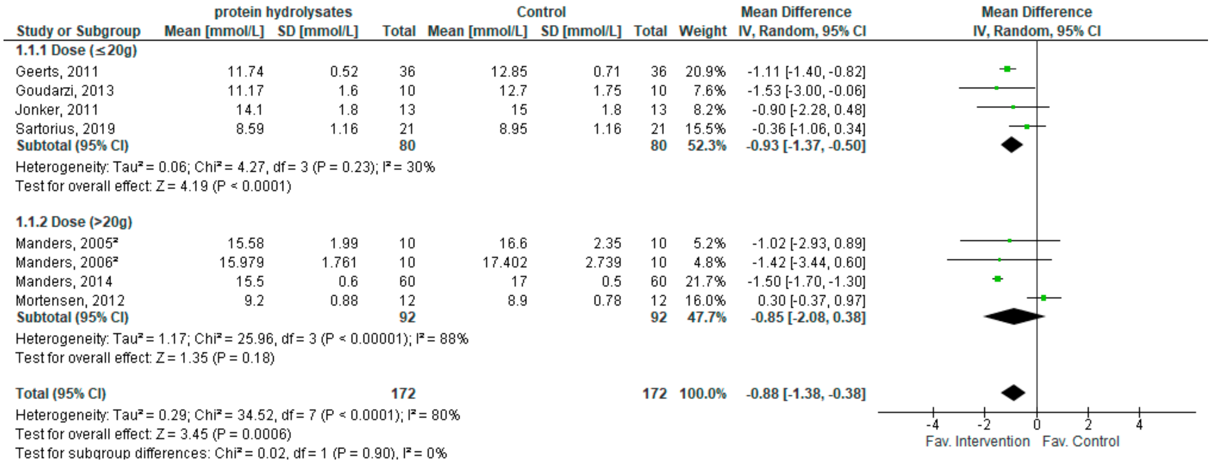

Figure S5. Subgroup analysis of acute studies investigating the effect of different doses of protein hydrolysates consumption on postprandial glucose response in adults with hyperglycaemia.

Study or Subgroup	protein hydrolysates Mean [mmol/L]	SD [mmol/L]	Total	Control Mean [mmol/L]	SD [mmol/L]	Total	Weight	Mean Difference IV, Random, 95% CI
Geerts, 2011	11.74	0.52	36	12.85	0.71	36	25.6%	-1.11 [-1.40, -0.82]
Goudarzi, 2013	11.17	1.6	10	12.7	1.75	10	10.1%	-1.53 [-3.00, -0.06]
Jonker, 2011	14.1	1.8	13	15	1.8	13	10.9%	-0.90 [-2.28, 0.48]
Manders, 2005ᵃ	15.58	1.99	10	16.6	2.35	10	7.1%	-1.02 [-2.93, 0.89]
Manders, 2006ᵃ	15.979	1.761	10	17.402	2.739	10	6.5%	-1.42 [-3.44, 0.60]
Mortensen, 2012	9.2	0.88	12	8.9	0.78	12	20.2%	0.30 [-0.37, 0.97]
Sartorius, 2019	8.59	1.16	21	8.95	1.16	21	19.7%	-0.36 [-1.06, 0.34]
**Total (95% CI)**			**112**			**112**	**100.0%**	**-0.71 [-1.30, -0.12]**
Heterogeneity: Tau² = 0.33; Chi² = 17.66, df = 6 (P = 0.007); I² = 66%								
Test for overall effect: Z = 2.37 (P = 0.02)								

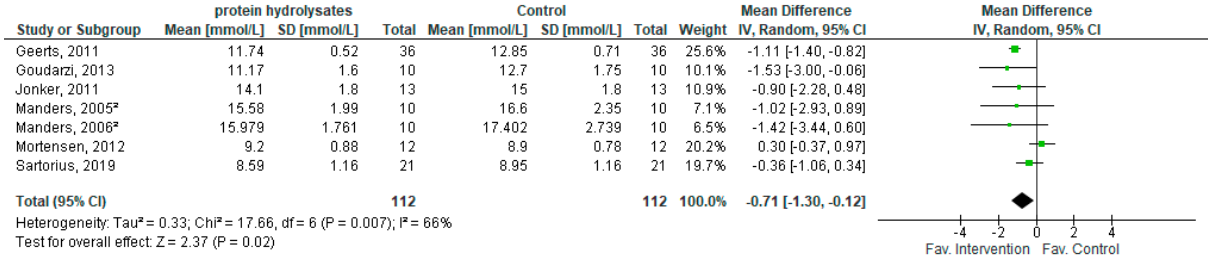

Figure S6. Sensitivity analysis by removing one study based on BMI of the studies investigating postprandial glucose response after protein hydrolysates consumption in adults with hyperglycaemia (Only studies BMI<30 Kg/m^2 were included in this analysis).

Study or Subgroup	protein hydrolysates Mean [mmol/L]	SD [mmol/L]	Total	Control Mean [mmol/L]	SD [mmol/L]	Total	Weight	Mean Difference IV, Random, 95% CI
Geerts, 2011	11.74	0.52	36	12.85	0.71	36	32.8%	-1.11 [-1.40, -0.82]
Jonker, 2011	14.1	1.8	13	15	1.8	13	6.6%	-0.90 [-2.28, 0.48]
Manders, 2005ᵃ	15.58	1.99	10	16.6	2.35	10	3.7%	-1.02 [-2.93, 0.89]
Manders, 2006ᵃ	15.979	1.761	10	17.402	2.739	10	3.4%	-1.42 [-3.44, 0.60]
Manders, 2014	15.5	0.6	60	17	0.5	60	36.2%	-1.50 [-1.70, -1.30]
Sartorius, 2019	8.59	1.16	21	8.95	1.16	21	17.3%	-0.36 [-1.06, 0.34]
**Total (95% CI)**			**150**			**150**	**100.0%**	**-1.11 [-1.50, -0.73]**
Heterogeneity: Tau² = 0.10; Chi² = 12.88, df = 5 (P = 0.02); I² = 61%								
Test for overall effect: Z = 5.64 (P < 0.00001)								

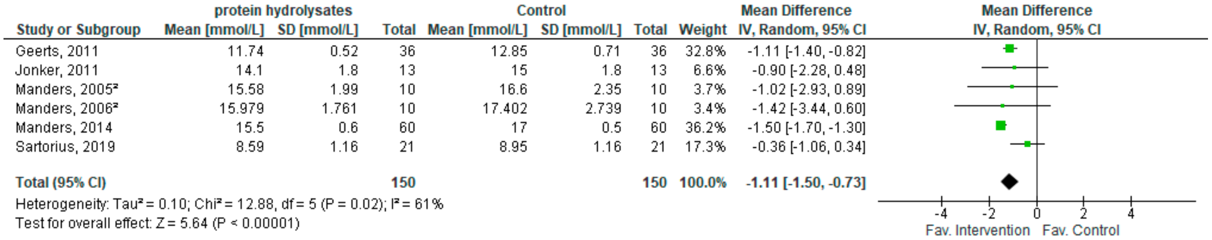

Figure S7. Sensitivity analysis by removing two studies based on control type of the studies investigating glucose response after protein hydrolysates consumption in adults with hyperglycaemia (Only studies with CHO control were included in this analysis).

Study or Subgroup	protein hydrolysates Mean [mmol/L]	SD [mmol/L]	Total	Control Mean [mmol/L]	SD [mmol/L]	Total	Weight	Mean Difference IV, Random, 95% CI
Geerts, 2011	11.74	0.52	36	12.85	0.71	36	22.4%	-1.11 [-1.40, -0.82]
Jonker, 2011	14.1	1.8	13	15	1.8	13	9.1%	-0.90 [-2.28, 0.48]
Manders, 2005[2]	15.58	1.99	10	16.6	2.35	10	5.8%	-1.02 [-2.93, 0.89]
Manders, 2006[2]	15.979	1.761	10	17.402	2.739	10	5.3%	-1.42 [-3.44, 0.60]
Manders, 2014	15.5	0.6	60	17	0.5	60	23.2%	-1.50 [-1.70, -1.30]
Mortensen, 2012	9.2	0.88	12	8.9	0.78	12	17.4%	0.30 [-0.37, 0.97]
Sartorius, 2019	8.59	1.16	21	8.95	1.16	21	16.9%	-0.36 [-1.06, 0.34]
**Total (95% CI)**			**162**			**162**	**100.0%**	**-0.82 [-1.35, -0.29]**

Heterogeneity: $Tau^2 = 0.31$; $Chi^2 = 34.36$, df = 6 (P < 0.00001); $I^2 = 83\%$
Test for overall effect: Z = 3.04 (P = 0.002)

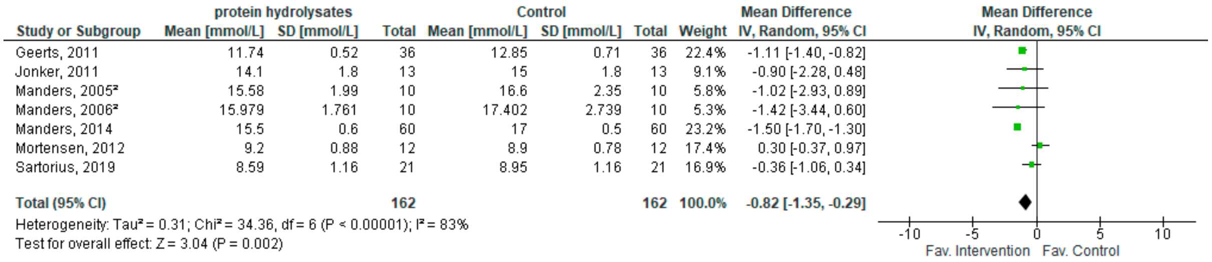

Figure S8. Sensitivity analysis by removing one study based on participants' age of the studies investigating postprandial glucose response after protein hydrolysates consumption in adults with hyperglycaemia (Only studies with older adulthood (> 55 years) were included in this analysis).

Study or Subgroup	Protein hydrolysates Mean	SD	Total	Control Mean	SD	Total	Weight	Mean Difference IV, Random, 95% CI
**1.2.1 CHO**								
Akhavan, 2010	36.6	27.9	21	42.4	13.98	21	11.0%	-5.80 [-19.15, 7.55]
Ballard, 2013	178	33.2	21	97.14	57.28	21	6.8%	80.86 [52.54, 109.18]
Claessens, 2007	35.77	7.67	8	38.62	21.85	8	10.2%	-2.85 [-18.90, 13.20]
Manders, 2006 (H)	141.59	78.12	10	85.56	37.79	10	3.0%	56.03 [2.24, 109.82]
**Subtotal (95% CI)**			**60**			**60**	**31.1%**	**28.17 [-8.91, 65.25]**
Heterogeneity: $Tau^2 = 1209.69$; $Chi^2 = 34.31$, df = 3 (P < 0.00001); $I^2 = 91\%$								
Test for overall effect: Z = 1.49 (P = 0.14)								
**1.2.2 Unhydrolysd protein**								
Bendtsen, 2014	102.8	12.54	24	99	11.2	24	12.7%	3.80 [-2.93, 10.53]
Calbet, 2002	86.18	44.79	6	55.9	18	6	4.8%	30.28 [-8.34, 68.90]
Dale, 2018	69.6	52.7	41	70.3	53.6	41	8.2%	-0.70 [-23.71, 22.31]
Drummond, 2018	63.99	48.8	62	53.38	37.8	62	10.4%	10.61 [-4.75, 25.97]
Horner, 2019	57.99	19.95	8	57.11	25.81	8	8.3%	0.88 [-21.73, 23.49]
Koopman, 2009	50.2	7.6	10	26.2	3.7	10	12.9%	24.00 [18.76, 29.24]
Power, 2009	43.06	17.87	16	33.69	15.08	16	11.5%	9.37 [-2.09, 20.83]
**Subtotal (95% CI)**			**167**			**167**	**68.9%**	**10.70 [0.83, 20.56]**
Heterogeneity: $Tau^2 = 110.74$; $Chi^2 = 27.08$, df = 6 (P = 0.0001); $I^2 = 78\%$								
Test for overall effect: Z = 2.13 (P = 0.03)								
**Total (95% CI)**			**227**			**227**	**100.0%**	**13.54 [2.92, 24.17]**

Heterogeneity: $Tau^2 = 220.26$; $Chi^2 = 63.59$, df = 10 (P < 0.00001); $I^2 = 84\%$
Test for overall effect: Z = 2.50 (P = 0.01)
Test for subgroup differences: $Chi^2 = 0.80$, df = 1 (P = 0.37), $I^2 = 0\%$

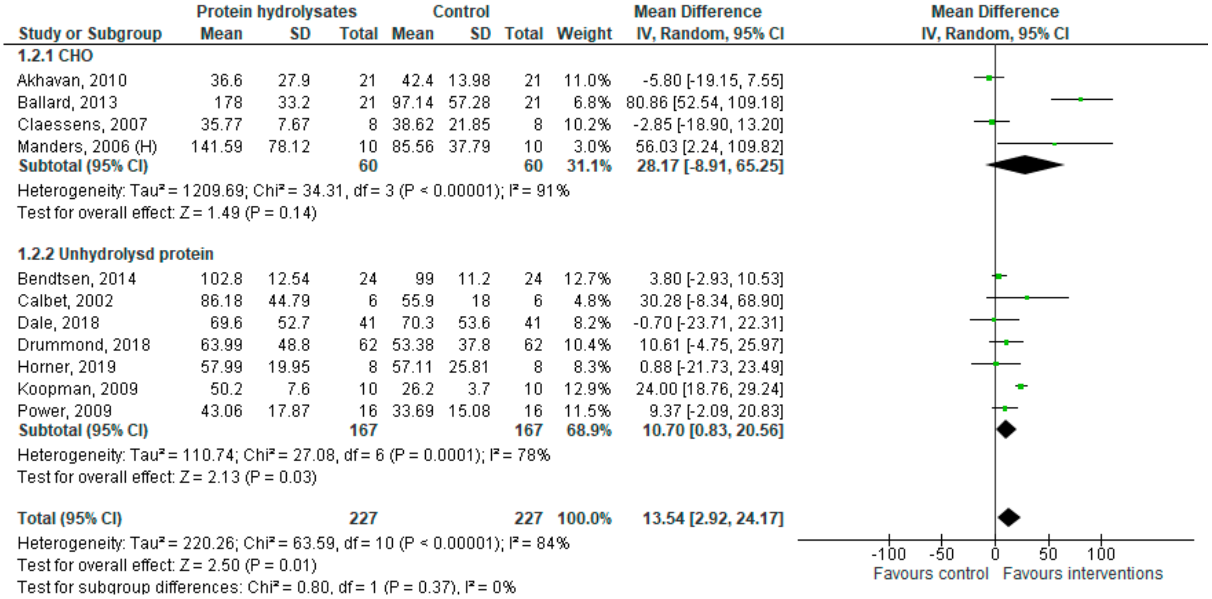

Figure S9. Subgroup analysis by control type on acute studies investigating postprandial insulin response after protein hydrolysates consumption in normoglycaemic adults

Study or Subgroup	Protein hydrolysates Mean [mIU/L]	SD [mIU/L]	Total	Control Mean [mIU/L]	SD [mIU/L]	Total	Weight	Mean Difference IV, Random, 95% CI
**1.2.1 BMI ≤ 25 Kg/m^2**								
Akhavan, 2010	36.6	27.9	21	42.4	13.98	21	10.5%	-5.80 [-19.15, 7.55]
Calbet, 2002	86.18	44.79	6	55.9	18	6	4.5%	30.28 [-8.34, 68.90]
Claessens, 2007	35.77	7.67	8	38.62	21.85	8	9.7%	-2.85 [-18.90, 13.20]
Dale, 2018	69.6	52.7	41	70.3	53.6	41	7.8%	-0.70 [-23.71, 22.31]
Koopman, 2009	50.2	7.6	10	26.2	3.7	10	12.4%	24.00 [18.76, 29.24]
Power, 2009	43.06	17.87	16	33.69	15.08	16	11.0%	9.37 [-2.09, 20.83]
Van-Loon, 2000	74.34	34.03	8	71.13	39.5	8	4.9%	3.21 [-32.92, 39.34]
**Subtotal (95% CI)**			**110**			**110**	**60.8%**	**7.58 [-4.94, 20.09]**
Heterogeneity: $Tau^2 = 187.83$; $Chi^2 = 28.81$, df = 6 (P < 0.0001); $I^2 = 79\%$								
Test for overall effect: Z = 1.19 (P = 0.24)								
**1.2.2 BMI > Kg/m^2**								
Ballard, 2013	178	33.2	21	97.14	57.28	21	6.5%	80.86 [52.54, 109.18]
Bendtsen, 2014	102.8	12.54	24	99	11.2	24	12.1%	3.80 [-2.93, 10.53]
Drummond, 2018	63.99	48.8	62	53.38	37.8	62	9.9%	10.61 [-4.75, 25.97]
Horner, 2019	57.99	19.95	8	57.11	25.81	8	7.9%	0.88 [-21.73, 23.49]
Manders, 2006[1]	141.59	78.12	10	85.56	37.79	10	2.8%	56.03 [2.24, 109.82]
**Subtotal (95% CI)**			**125**			**125**	**39.2%**	**25.06 [1.75, 48.37]**
Heterogeneity: $Tau^2 = 540.04$; $Chi^2 = 30.47$, df = 4 (P < 0.00001); $I^2 = 87\%$								
Test for overall effect: Z = 2.11 (P = 0.04)								
**Total (95% CI)**			**235**			**235**	**100.0%**	**12.98 [2.73, 23.23]**

Heterogeneity: $Tau^2 = 214.20$; $Chi^2 = 63.91$, df = 11 (P < 0.00001); $I^2 = 83\%$
Test for overall effect: Z = 2.48 (P = 0.01)
Test for subgroup differences: $Chi^2 = 1.68$, df = 1 (P = 0.20), $I^2 = 40.4\%$

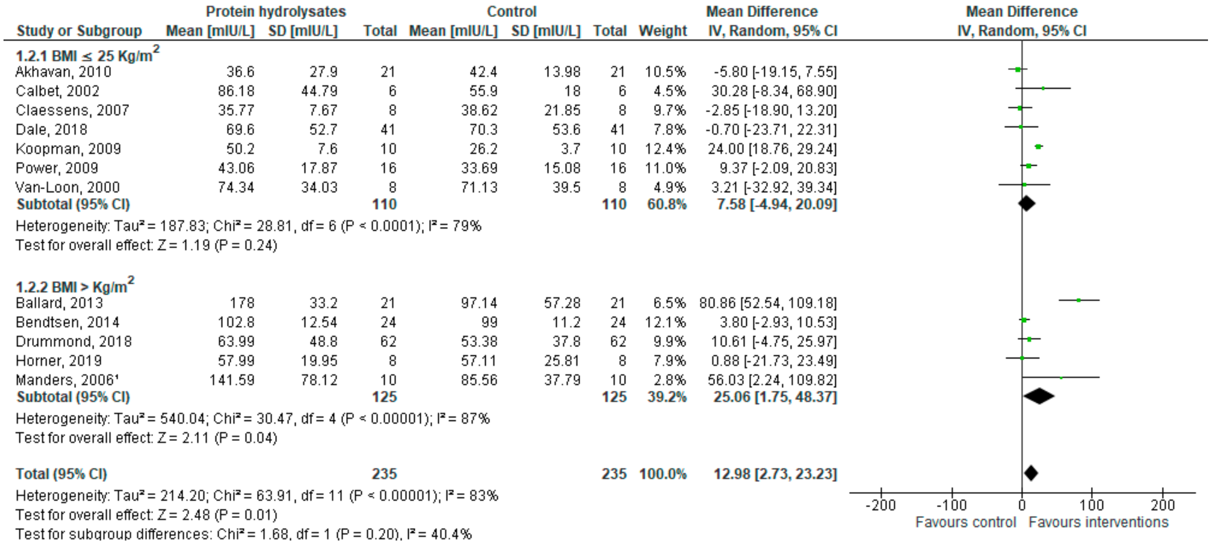

Figure S10. Subgroup analysis by BMI on acute studies investigating postprandial insulin response after protein hydrolysates consumption in adults with normoglycaemia.

	Protein hydrolysates			Control				Mean Difference
**Study or Subgroup**	**Mean [mIU/L]**	**SD [mIU/L]**	**Total**	**Mean [mIU/L]**	**SD [mIU/L]**	**Total**	**Weight**	**IV, Random, 95% CI**
**1.2.1 Dose ≤20g**								
Akhavan, 2010	36.6	27.9	21	42.4	13.98	21	9.9%	-5.80 [-19.15, 7.55]
Ballard, 2013	178	33.2	21	97.14	57.28	21	6.0%	80.86 [52.54, 109.18]
Calbet, 2002	86.18	44.79	6	55.9	18	6	4.2%	30.28 [-8.34, 68.90]
Dale, 2018	69.6	52.7	41	70.3	53.6	41	7.3%	-0.70 [-23.71, 22.31]
Drummond, 2018	63.99	48.8	62	53.38	37.8	62	9.4%	10.61 [-4.75, 25.97]
Horner, 2019	57.99	19.95	8	57.11	25.81	8	7.4%	0.88 [-21.73, 23.49]
**Subtotal (95% CI)**			**159**			**159**	**44.2%**	**17.16 [-4.57, 38.88]**
Heterogeneity: Tau^2 = 589.95; Chi^2 = 31.84, df = 5 (P < 0.00001); I^2 = 84%								
Test for overall effect: Z = 1.55 (P = 0.12)								
**1.2.3 Dose > 20g**								
Agergaard, 2021	59.488	35.38	12	51.12	38.65	12	5.8%	8.37 [-21.28, 38.01]
Bendtsen, 2014	102.8	12.54	24	99	11.2	24	11.5%	3.80 [-2.93, 10.53]
Claessens, 2007	35.77	7.67	8	38.62	21.85	8	9.2%	-2.85 [-18.90, 13.20]
Koopman, 2009	50.2	7.6	10	26.2	3.7	10	11.7%	24.00 [18.76, 29.24]
Manders, 2006'	141.59	78.12	10	85.56	37.79	10	2.6%	56.03 [2.24, 109.82]
Power, 2009	43.06	17.87	16	33.69	15.08	16	10.4%	9.37 [-2.09, 20.83]
Van-Loon, 2000	74.34	34.03	8	71.13	39.5	8	4.6%	3.21 [-32.92, 39.34]
**Subtotal (95% CI)**			**88**			**88**	**55.8%**	**10.72 [-0.56, 22.00]**
Heterogeneity: Tau^2 = 137.94; Chi^2 = 30.44, df = 6 (P < 0.0001); I^2 = 80%								
Test for overall effect: Z = 1.86 (P = 0.06)								
**Total (95% CI)**			**247**			**247**	**100.0%**	**12.65 [2.84, 22.46]**
Heterogeneity: Tau^2 = 206.09; Chi^2 = 64.03, df = 12 (P < 0.00001); I^2 = 81%								
Test for overall effect: Z = 2.53 (P = 0.01)								
Test for subgroup differences: Chi^2 = 0.27, df = 1 (P = 0.61), I^2 = 0%								

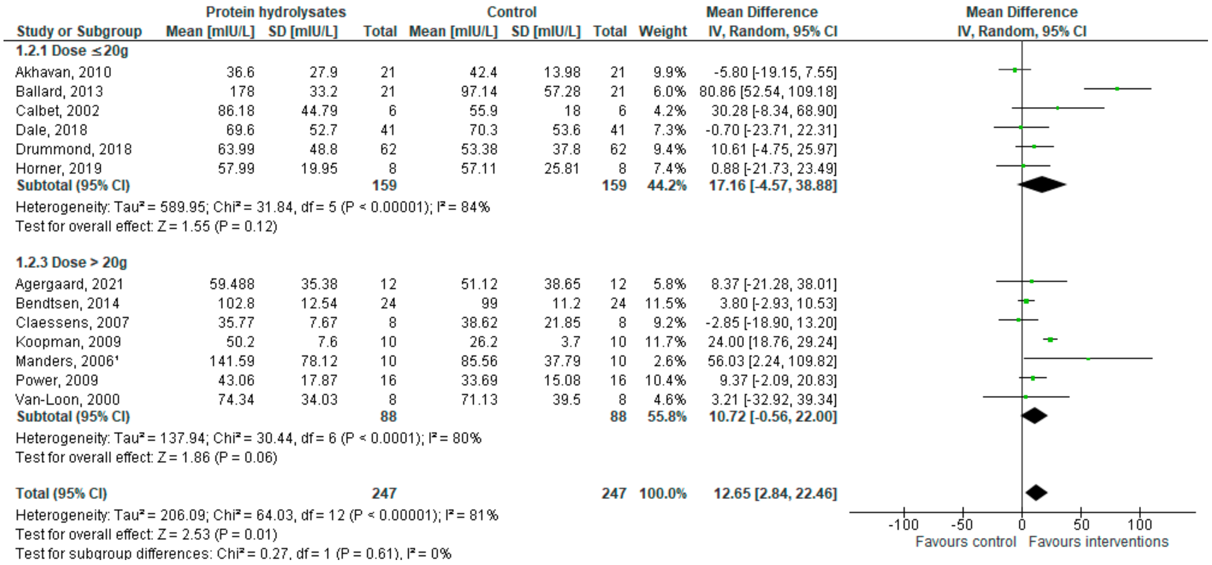

Figure S11. Subgroup analysis of acute studies investigating the effect of different doses of protein hydrolysates consumption on postprandial insulin response in adults with normoglycaemia.

	Protein hydrolysates			Control				Mean Difference
**Study or Subgroup**	**Mean [mIU/L]**	**SD [mIU/L]**	**Total**	**Mean [mIU/L]**	**SD [mIU/L]**	**Total**	**Weight**	**IV, Random, 95% CI**
**1.2.1 18 to 35 y**								
Akhavan, 2010	36.6	27.9	21	42.4	13.98	21	9.9%	-5.80 [-19.15, 7.55]
Bendtsen, 2014	102.8	12.54	24	99	11.2	24	11.5%	3.80 [-2.93, 10.53]
Calbet, 2002	86.18	44.79	6	55.9	18	6	4.2%	30.28 [-8.34, 68.90]
Claessens, 2007	35.77	7.67	8	38.62	21.85	8	9.2%	-2.85 [-18.90, 13.20]
Power, 2009	43.06	17.87	16	33.69	15.08	16	10.4%	9.37 [-2.09, 20.83]
Van-Loon, 2000	74.34	34.03	8	71.13	39.5	8	4.6%	3.21 [-32.92, 39.34]
**Subtotal (95% CI)**			**83**			**83**	**49.8%**	**3.25 [-2.12, 8.62]**
Heterogeneity: Tau^2 = 3.24; Chi^2 = 5.32, df = 5 (P = 0.38); I^2 = 6%								
Test for overall effect: Z = 1.19 (P = 0.24)								
**1.2.2 36 to 55 y**								
Ballard, 2013	178	33.2	21	97.14	57.28	21	6.0%	80.86 [52.54, 109.18]
Dale, 2018	69.6	52.7	41	70.3	53.6	41	7.3%	-0.70 [-23.71, 22.31]
Drummond, 2018	63.99	48.8	62	53.38	37.8	62	9.4%	10.61 [-4.75, 25.97]
**Subtotal (95% CI)**			**124**			**124**	**22.7%**	**29.05 [-12.95, 71.06]**
Heterogeneity: Tau^2 = 1244.50; Chi^2 = 22.28, df = 2 (P < 0.0001); I^2 = 91%								
Test for overall effect: Z = 1.36 (P = 0.18)								
**1.2.3 56 y and older**								
Agergaard, 2021	59.488	35.38	12	51.12	38.65	12	5.8%	8.37 [-21.28, 38.01]
Horner, 2019	57.99	19.95	8	57.11	25.81	8	7.4%	0.88 [-21.73, 23.49]
Koopman, 2009	50.2	7.6	10	26.2	3.7	10	11.7%	24.00 [18.76, 29.24]
Manders, 2006'	141.59	78.12	10	85.56	37.79	10	2.6%	56.03 [2.24, 109.82]
**Subtotal (95% CI)**			**40**			**40**	**27.5%**	**17.67 [1.72, 33.61]**
Heterogeneity: Tau^2 = 128.64; Chi^2 = 6.19, df = 3 (P = 0.10); I^2 = 52%								
Test for overall effect: Z = 2.17 (P = 0.03)								
**Total (95% CI)**			**247**			**247**	**100.0%**	**12.65 [2.84, 22.46]**
Heterogeneity: Tau^2 = 206.09; Chi^2 = 64.03, df = 12 (P < 0.00001); I^2 = 81%								
Test for overall effect: Z = 2.53 (P = 0.01)								
Test for subgroup differences: Chi^2 = 4.09, df = 2 (P = 0.13), I^2 = 51.1%								

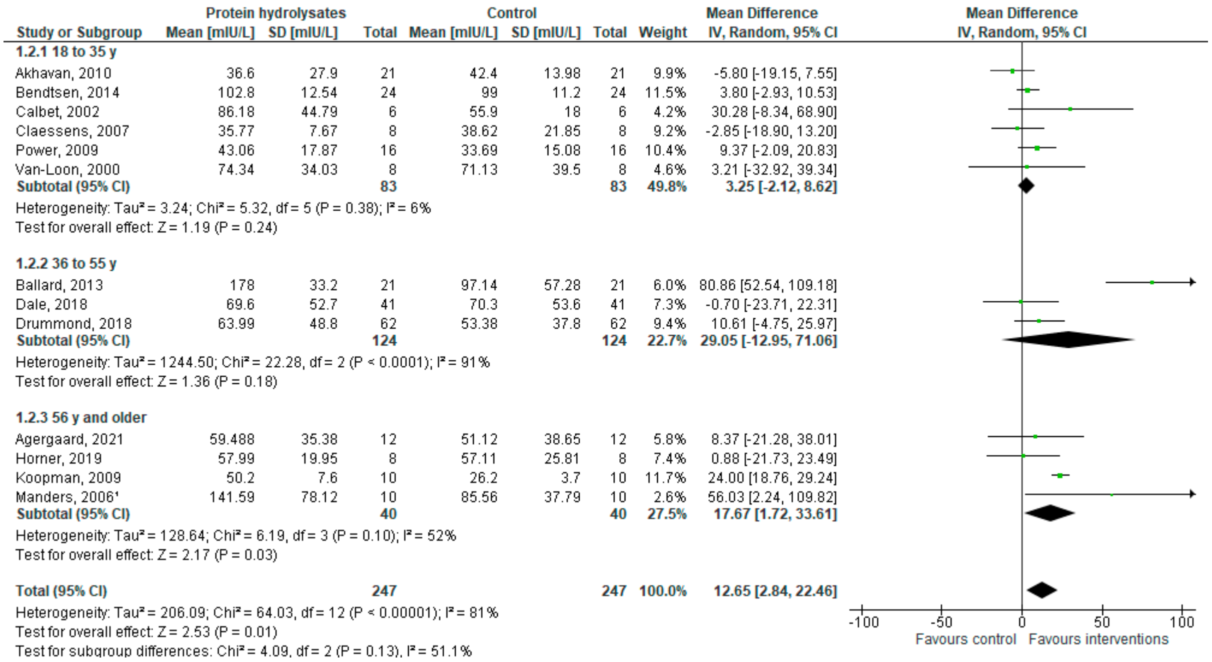

Figure S12. Subgroup analysis of acute studies investigating the impact of the participants' age on the postprandial insulin in response to protein hydrolysates consumption in adults with normoglycaemia.

Study or Subgroup	Protein hydrolysates Mean [mIU/L]	SD [mIU/L]	Total	Control Mean [mIU/L]	SD [mIU/L]	Total	Weight	Mean Difference IV, Random, 95% CI
**1.2.1 Dose ≤ 20g**								
Geerts, 2011	30.76	17.34	36	20.11	17.34	36	18.9%	10.65 [2.64, 18.66]
Goudarzi, 2013	72.83	13.93	10	40.48	9.88	10	18.2%	32.35 [21.77, 42.93]
Jonker, 2011	53.6	46.5	13	42.6	35.7	13	10.8%	11.00 [-20.87, 42.87]
Sartorius, 2019	76.84	31.91	21	78.074	31.91	21	15.2%	-1.23 [-20.54, 18.07]
**Subtotal (95% CI)**			**80**			**80**	**63.2%**	**14.60 [-0.85, 30.04]**
Heterogeneity: Tau² = 174.45; Chi² = 14.02, df = 3 (P = 0.003); I² = 79%								
Test for overall effect: Z = 1.85 (P = 0.06)								
**1.2.2 Dose > 20g**								
Manders, 2006²	84.05	34.3	10	33.1	25.72	10	12.6%	50.95 [24.38, 77.52]
Manders, 2014	116.3	71.26	60	61.3	41.83	60	14.6%	55.00 [34.09, 75.91]
Mortensen, 2012	59.6	40.5	11	62.7	45.1	11	9.7%	-3.10 [-38.92, 32.72]
**Subtotal (95% CI)**			**81**			**81**	**36.8%**	**37.13 [5.76, 68.49]**
Heterogeneity: Tau² = 568.16; Chi² = 7.97, df = 2 (P = 0.02); I² = 75%								
Test for overall effect: Z = 2.32 (P = 0.02)								
**Total (95% CI)**			**161**			**161**	**100.0%**	**23.05 [7.53, 38.57]**
Heterogeneity: Tau² = 315.12; Chi² = 32.99, df = 6 (P < 0.0001); I² = 82%								
Test for overall effect: Z = 2.91 (P = 0.004)								
Test for subgroup differences: Chi² = 1.60, df = 1 (P = 0.21), I² = 37.3%								

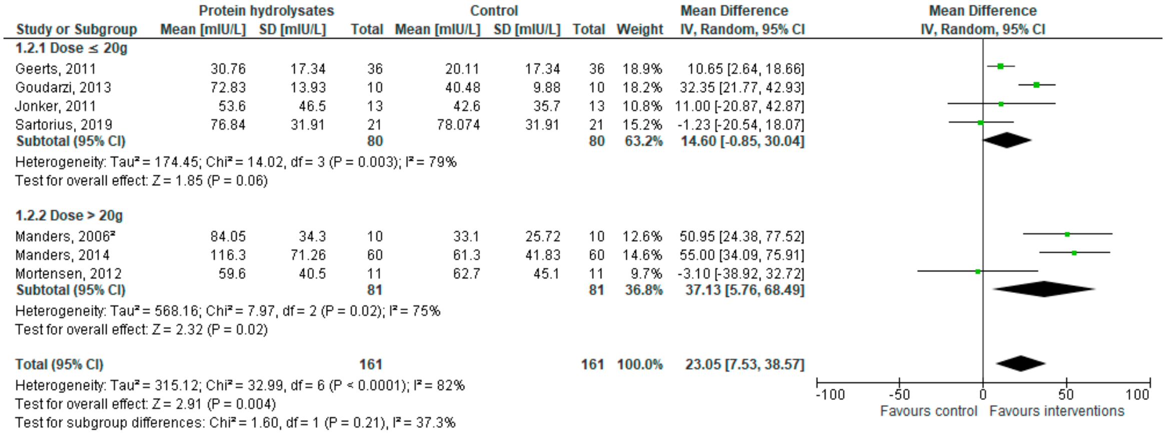

Figure S13. Subgroup analysis of acute studies investigating the effect of different doses of protein hydrolysates consumption on postprandial insulin response in adults with hyperglycaemia.

Study or Subgroup	Protein hydrolysates Mean [mIU/L]	SD [mIU/L]	Total	Control Mean [mIU/L]	SD [mIU/L]	Total	Weight	Mean Difference IV, Random, 95% CI
Geerts, 2011	30.76	17.34	36	20.11	17.34	36	24.4%	10.65 [2.64, 18.66]
Jonker, 2011	53.6	46.5	13	42.6	35.7	13	16.1%	11.00 [-20.87, 42.87]
Manders, 2006²	84.05	34.3	10	33.1	25.72	10	18.1%	50.95 [24.38, 77.52]
Manders, 2014	116.3	71.26	60	61.3	41.83	60	20.3%	55.00 [34.09, 75.91]
Sartorius, 2019	76.84	31.91	21	78.074	31.91	21	20.9%	-1.23 [-20.54, 18.07]
**Total (95% CI)**			**140**			**140**	**100.0%**	**24.55 [3.30, 45.80]**
Heterogeneity: Tau² = 464.30; Chi² = 24.88, df = 4 (P < 0.0001); I² = 84%								
Test for overall effect: Z = 2.26 (P = 0.02)								

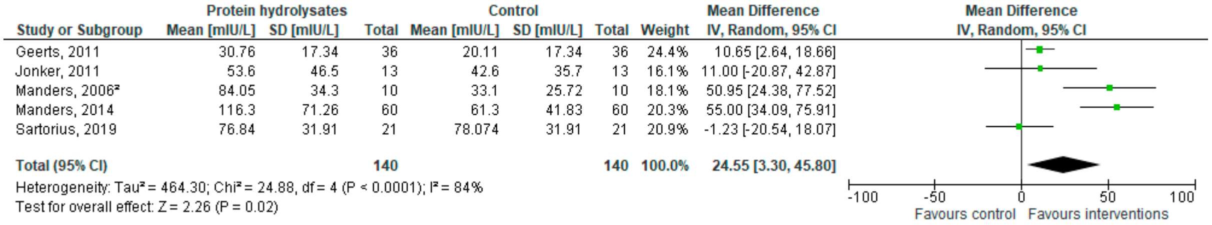

Figure S14. Sensitivity analysis by removing two studies based on control type of the studies investigating postprandial insulin response after protein hydrolysates consumption in adults with hyperglycaemia (Only studies with CHO control were included in this analysis).

Study or Subgroup	Protein hydrolysates Mean [mIU/L]	SD [mIU/L]	Total	Control Mean [mIU/L]	SD [mIU/L]	Total	Weight	Mean Difference IV, Random, 95% CI
Geerts, 2011	30.76	17.34	36	20.11	17.34	36	23.8%	10.65 [2.64, 18.66]
Goudarzi, 2013	72.83	13.93	10	40.48	9.88	10	22.6%	32.35 [21.77, 42.93]
Jonker, 2011	53.6	46.5	13	42.6	35.7	13	11.7%	11.00 [-20.87, 42.87]
Manders,2006²	84.05	34.3	10	33.1	25.72	10	14.0%	50.95 [24.38, 77.52]
Mortensen, 2012	59.6	40.5	11	62.7	45.1	11	10.2%	-3.10 [-38.92, 32.72]
Sartorius, 2019	76.84	31.91	21	78.074	31.91	21	17.8%	-1.23 [-20.54, 18.07]
**Total (95% CI)**			**101**			**101**	**100.0%**	**17.71 [2.96, 32.46]**
Heterogeneity: Tau² = 221.49; Chi² = 21.51, df = 5 (P = 0.0006); I² = 77%								
Test for overall effect: Z = 2.35 (P = 0.02)								

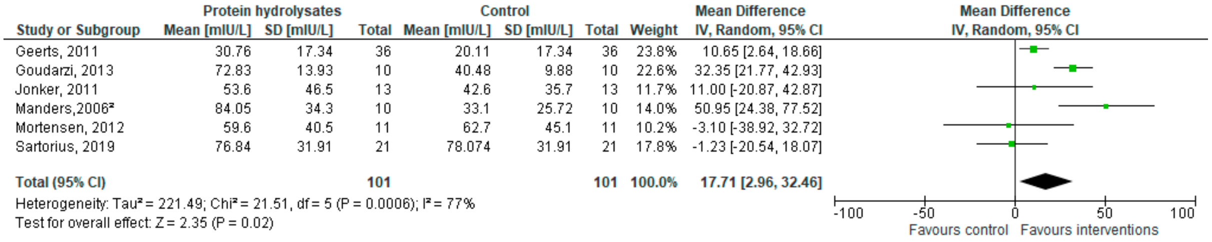

Figure S15. Sensitivity analysis by removing one study based on BMI of the studies investigating postprandial insulin response after protein hydrolysates consumption in adults with hyperglycaemia (Only studies BMI < 30 Kg/m² were included in this analysis).

Study or Subgroup	Protein hydrolysates Mean [mIU/L]	SD [mIU/L]	Total	Control Mean [mIU/L]	SD [mIU/L]	Total	Weight	Mean Difference IV, Random, 95% CI
Geerts, 2011	30.76	17.34	36	20.11	17.34	36	21.7%	10.65 [2.64, 18.66]
Jonker, 2011	53.6	46.5	13	42.6	35.7	13	13.9%	11.00 [-20.87, 42.87]
Manders, 2006²	84.05	34.3	10	33.1	25.72	10	15.7%	50.95 [24.38, 77.52]
Manders, 2014	116.3	71.26	60	61.3	41.83	60	17.8%	55.00 [34.09, 75.91]
Mortensen, 2012	59.6	40.5	11	62.7	45.1	11	12.6%	-3.10 [-38.92, 32.72]
Sartorius, 2019	76.84	31.91	21	78.074	31.91	21	18.3%	-1.23 [-20.54, 18.07]
**Total (95% CI)**			**151**			**151**	**100.0%**	**21.00 [1.82, 40.18]**
Heterogeneity: Tau² = 425.21; Chi² = 25.96, df = 5 (P < 0.0001); I² = 81%								
Test for overall effect: Z = 2.15 (P = 0.03)								

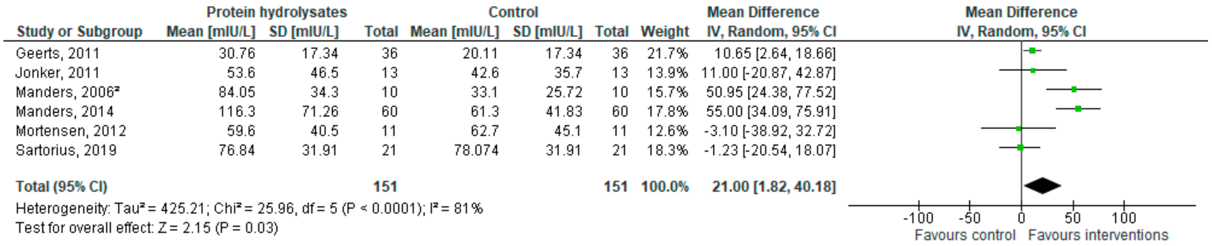

Figure S16. Sensitivity analysis by removing one study based on participants' age of the studies investigating postprandial insulin response after protein hydrolysates consumption in adults with hyperglycaemia (Only studies with older adulthood (>55 years) were included in this analysis).

Study or Subgroup	Experimental Mean [mmol/L]	Experimental SD [mmol/L]	Experimental Total	Control Mean [mmol/L]	Control SD [mmol/L]	Control Total	Weight	Mean Difference IV, Random, 95% CI
**1.1.1 Normoglycaemia**								
Ballard, 2009	5.21	0.42	20	5.19	0.39	20	22.7%	0.02 [-0.23, 0.27]
Rakvaag, 2019 (ah)	5.6	0.4	15	5.8	0.4	16	22.5%	-0.20 [-0.48, 0.08]
Rakvaag, 2019 (al)	5.7	0.6	17	5.6	0.6	17	21.4%	0.10 [-0.30, 0.50]
**Subtotal (95% CI)**			**52**			**53**	**66.5%**	**-0.05 [-0.22, 0.12]**
Heterogeneity: Tau² = 0.00; Chi² = 1.92, df = 2 (P = 0.38); I² = 0%								
Test for overall effect: Z = 0.53 (P = 0.60)								
**1.1.2 Hyperglycaemia**								
Devesia, 2020 (ah)	6.1	0.9	21	9.2	1.9	10	11.8%	-3.10 [-4.34, -1.86]
Devesia, 2020 (al)	10.8	3.4	20	11.3	3	10	5.1%	-0.50 [-2.88, 1.88]
Zue, 2010	6.59	1.56	50	8.5	2.47	50	16.6%	-1.91 [-2.72, -1.10]
**Subtotal (95% CI)**			**91**			**70**	**33.5%**	**-2.10 [-3.24, -0.96]**
Heterogeneity: Tau² = 0.54; Chi² = 4.44, df = 2 (P = 0.11); I² = 55%								
Test for overall effect: Z = 3.62 (P = 0.0003)								
**Total (95% CI)**			**143**			**123**	**100.0%**	**-0.73 [-1.33, -0.12]**
Heterogeneity: Tau² = 0.40; Chi² = 43.25, df = 5 (P < 0.00001); I² = 88%								
Test for overall effect: Z = 2.36 (P = 0.02)								
Test for subgroup differences: Chi² = 12.24, df = 1 (P = 0.0005), I² = 91.8%								

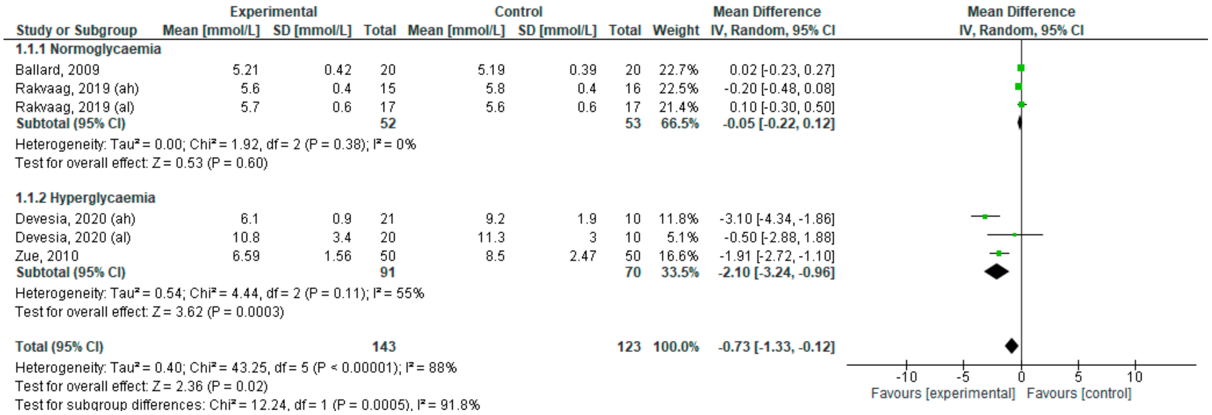

Figure S17. Subgroup analysis of long-term studies investigating the impact of the glycaemic status of the participants on fasting glucose levels in response to protein hydrolysates consumption.
